# Comparative transcriptomic, epigenomic and immunological analyses identify drivers of disparity in high-grade serous ovarian cancer

**DOI:** 10.1038/s41525-024-00448-2

**Published:** 2024-12-02

**Authors:** Hao Huang, Russel Keathley, Ujin Kim, Horacio Cardenas, Ping Xie, Jianjun Wei, Ernst Lengyel, Kenneth P. Nephew, Guangyuan Zhao, Zhen Fu, Emma L. Barber, Masha Kocherginsky, Victoria Bae-Jump, Bin Zhang, Daniela Matei

**Affiliations:** 1 Department of Obstetrics and Gynecology, Feinberg School of Medicine, Northwestern University, Chicago, IL USA; 2 Department of Medicine; Hematology/Oncology Division, Feinberg School of Medicine, Northwestern University, Chicago, IL USA; 3 Department of Pathology, Feinberg School of Medicine, Northwestern University, Chicago, IL USA; 4 Department of Obstetrics and Gynecology/Section of Gynecologic Oncology, University of Chicago, Chicago, IL USA; 5 School of Medicine, Indiana University, Bloomington, IN USA; 6 Bioinformatics and Biostatistics Core, Van Andel Institute, Grand Rapids, MI USA; 7 Department of Preventive Medicine (Biostatistics), Feinberg School of Medicine, Northwestern University, Chicago, IL USA; 8 Robert H. Lurie Comprehensive Cancer Center, Chicago, IL USA; 9 Division of Gynecologic Oncology, University of North Carolina, Chapel Hill, NC USA; 10 Jesse Brown VA Medical Center, Chicago, IL USA

**Keywords:** Molecular medicine, Ovarian cancer, Cancer genomics

## Abstract

Black women face the highest mortality-to-incidence ratio from high grade serous ovarian cancer (HGSOC). This study investigated biological differences in HGSOC tumors from Black vs. White women. HGSOC from 35 Black and 31 White patients were analyzed by Infinium Methyation-EPIC array and RNA sequencing. 191 CpG sites were differentially methylated (FDR < 0.05, β value change> 10%) and 277 genes were differentially expressed (FDR < 0.05). Gene Ontology identified enriched pathways related to DNA damage response, p53/apoptosis signaling, and cholesterol/lipid metabolism directly connected with genes like *INSR*, *FOXA1* and *FOXB1*. *INSR* and *FOXA1* knockdown enhanced cisplatin sensitivity and inhibited cell proliferation and colony formation. Tumors from Black patients were infiltrated by fewer CD4+ naïve and regulatory T-cells. Overall, differences in DNA methylation, transcriptomic profiles and immune cell infiltration were detected in tumors from Black vs. White patients. Further investigation is warranted into how these differences may affect treatment response and outcomes in Black women.

## Introduction

Epithelial ovarian cancer (OC) is the most lethal gynecological cancer with a lifetime risk of 1.3%^1^. High-grade serous ovarian cancer (HGSOC) is the most common subtype, accounting for approximately 70% of all OC cases. HGSOC originates from the epithelium of the fallopian tube and is often diagnosed at a late stage^2^. Loss of function *TP53* mutations are among the earliest oncogenic events in HGSOC development^2^. Treatment consists typically of platinum-based chemotherapy and cytoreductive surgery. While most patients are initially responsive to platinum based therapy, the median time to disease recurrence is 18–24 months. Among the challenges related to treatment of HGSOC are the high tumor heterogeneity, marked by variable gene expression and methylation, frequent p53 mutations and a high rate of copy number alterations leading to genomic instability^2^.

While significant advancements have been made in understanding HGSOC biology, substantial disparities in outcomes persist, particularly between Black and White women. Despite a higher incidence of OC in White women, the mortality rate for Black women is 1.3 times higher^3^. The mortality/incidence ratio among Black women is 0.68—the highest among all ethnic groups^4^. The disparities in disease presentation and outcomes can be attributed to various socioeconomic factors including barriers to healthcare access, environmental risk factors, higher rates of co-morbidities, obesity, and disparities in the quality of care received. Black women are more likely to have treatment delays or discontinuation contributing to the worst overall mortality among all ethnic groups analyzed^5^. Interestingly, in a retrospective analysis including 163 White and 47 Black women all treated at academic center, no disparity in survival was observed, underscoring the significance of treatment delivery and treatment quality to achieving equal clinical outcomes^6^.

However, recent research highlights the potential role of biological differences in health disparities across populations. A study of endometrial carcinomas profiled by The Cancer Genome Atlas (TCGA) found significant and broad differential methylation between tumors from Black and White women, with a negative correlation with gene expression among these probes^7^. Another study in endometrial cancer found differential ribosomal DNA methylation in tumors from Black vs. White patients^8^. Furthermore, ancestry has been linked to distinct tumor molecular features, supporting the idea that cancer genomics could be influenced by ancestry^9,10^. An analysis among a pan-cohort of patients with ovarian, cervical, uterine, or breast cancer from TCGA identified only 70 differentially expressed transcripts among Black and European American patient samples (2+ log-fold change)^11^. Pathway analysis in that group indicated changes in gene networks related to microtubule assembly and ciliated motility. Moreover, there were 61 differentially methylated probes between the two groups, and gene set enrichment identified “*metastasis*” and “*cadherin signaling*” among the gene networks associated with differentially methylated probes (DMPs). Further understanding of biological drivers of aggressive cancer phenotypes leading to outcome disparities in HGSOC is needed.

Here, we analyzed methylomic and transcriptomic profiles of 66 HGSOC tumors from Black or Non-Hispanic White women. We validated and characterized distinct features unique to each cohort and noted clinically relevant signatures. We studied the functions of two target genes, INSR and FOXA1, which were significantly upregulated in tumors from Black patients. Finally, we described differences in immune cell-type infiltration and enriched immune signatures between the two cohorts.

## Results

### Tumors from Black and White women display distinct differences in survival, methylomes, and transcriptomes

**Fig. 1:**
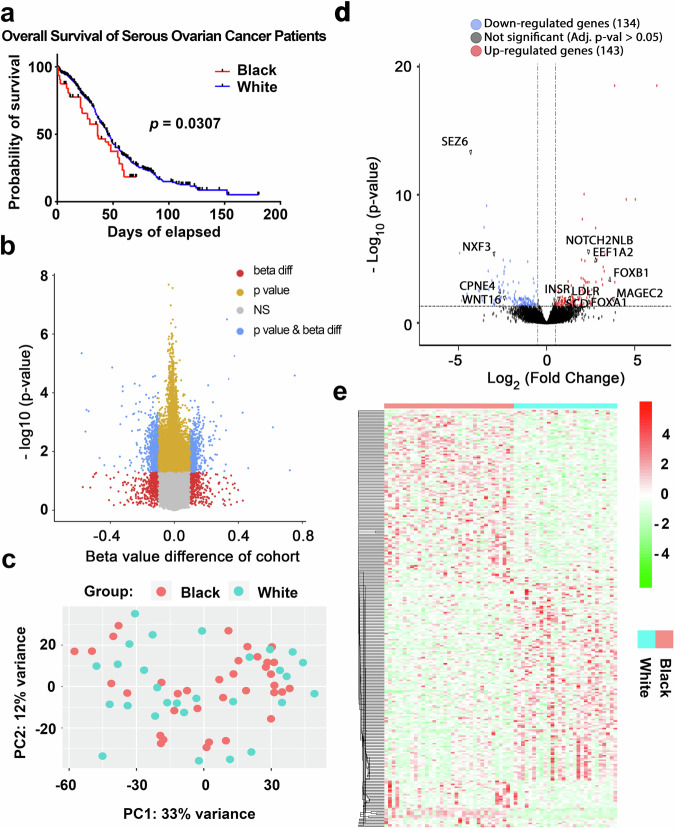
Comparative methylomic and RNA-Seq analysis of ovarian tumors from Black and White patients. **a** Kaplan-Meier survival curve shows the survival proportions of ovarian cancer patients over time differentiated by Black and White cohorts in the TCGA OC dataset (Nature 2011). (37 Black and 531 White; *p* = 0.0307). **b** Volcano plot of differentially methylated probes between Black and White cohorts. The plot visualizes the relationship between the magnitude of change in DNA methylation (beta value difference) on the x-axis and the statistical significance of these changes on the y-axis, measured as -log10(p-value). Red dots represent probes with beta values > 0.1, but non-significant p-values (*n* = 2,218). Yellow dots indicate probes with adjusted p-values < 0.05 (*n* = 10,713). Gray dots are probes that do not reach significance (NS), while blue dots represent probes that are significant by both criteria: beta value > 0.1 and adjusted p-value < 0.05 (*n* = 191). **c** Principal Component Analysis (PCA) of gene expression data from all samples (n = 63 patients, k = 22,511 gene-features). **d** Volcano plot of differentially expressed genes provides a visualization of genes up-regulated (right) and down-regulated (left) with significance levels plotted against the magnitude of change. Key genes with substantial changes are labeled. The dotted vertical lines indicate the fold change threshold (e.g., ±2-fold), with genes beyond these lines (i.e., farther to the right or left) being classified as up- or down-regulated. The horizontal dotted line marks the threshold for statistical significance (e.g., Adj. p-value = 0.05), with genes above this line being deemed statistically significantly differentially expressed. **e** A heatmap presents gene expression variations observed in the two groups, with color intensity indicating the level of variance. Light blue represents the White patients group, and red represents the Black patients group.

We used the TCGA publicly available dataset^12,13^ to validate the previously reported survival differences between Black and White women with HGSOC^1,3^. Kaplan-Meier survival curves (Fig. 1a) show that Black patients (*n* = 37) had a significant shorter survival (median OS 35.80 months) compared to White patients (*n* = 531, median OS 44.55 months). Given that Black patients exhibit shorter survival outcomes compared to White patients, we hypothesized that tumors from Black women harbor distinct epigenetic or immunological alterations, potentially contributing to a more aggressive disease course and unfavorable response to treatment. To test this hypothesis, we conducted an integrative genomic analysis, including DNA methylation profiling using Infinium Methyation-EPIC arrays and RNA-sequencing (RNA-Seq) in HGSOC tumor specimens from 35 Black and 31 White patients collected from several institutions. Patients’ characteristics are included in Supplementary Table 1. Patients in the two groups were of similar ages, but Black patients tended to have higher median BMI (31.00 vs. 26.56, *p* = 0.06) compared to White patients (Supplementary Table 2).

There was no clear separation of tumors from Black vs. White patients based on methylomic profiles as assessed by Principal Component Analysis (PCA, Supplementary Fig. 1a). However, there were 10,713 differentially methylated regions (DMRs, adjusted *p*-value < 0.05) illustrated in the Volcano plot in Fig. 1b. Several chromosomal regions were differentially methylated (in particular, regions located on chromosomes, 4, 6, 17). Of the DMRs, 191 regions were significantly differentially methylated (FDR < 0.05) and harbored a difference in β value greater than 10%. These regions corresponded to 78 genes (Supplementary Table 3). Pathway enrichment analysis of the genes associated with DMRs identified the histone deacetylase HDAC4 as being hypomethylated in tumors from Black patients and connected to pathways related to “*ATM signaling”, “Initiation of Transcription”, “Apelin signaling”* and *“CAMKK2 pathway*” (Supplementary Fig. 1b). Likewise, TNF superfamily member 11 (TNSF11), hypermethylated in tumors from Black women, was connected to networks related to “*lymphocyte activation” “leukocyte cell-cell adhesion” and “collagen synthesis”* (Supplementary Fig. 1c).

We next compared the transcriptomes of HGSOC tumors from Black (*n* = 35) vs White (*n* = 28) women using RNAseq. Dimensionality reduction with PCA assessed the overall similarity of samples within the same patient cohort and showed considerable overlap between the transcriptome of tumors from the two groups and limited clustering (PC1 = 33%) of patient cohort samples (Fig. 1c). The distribution of all genes, including non-significant and differentially expressed genes (DEGs) in Black vs. White cohorts, is shown in the volcano plot (Fig. 1d). Among the DEGs, 143 were upregulated (103 protein-coding) such as *MAGE family member C2 (MAGEC2), FOXB1, Notch 2 N-terminal like B (NOTCH2NLB), Encodes eukaryotic translation elongation (EEF1A2), FOXA1, Stearoyl-CoA desaturase (SCD), low density lipoprotein receptor (LDLR)*, and *Insulin receptor (INSR)*. Conversely, 134 genes (81 protein-coding) were downregulated in Black patients, including Seizure related 6 homologs *(SEZ6), Nuclear RNA export factor 3 (NXF3), Copine-4 (CPNE4)*, and *WNT16 (Wnt Family Member 16)* (Supplementary Table 4 and Supplementary Table 5). Distinct patterns of expression of the 277 DEGs between the Black and White patients are highlighted in the heatmap (Fig. 1e).

### Pathway enrichment analysis reveals distinct differences in molecular mechanisms engaged in tumors from Black vs. White patients

**Fig. 2:**
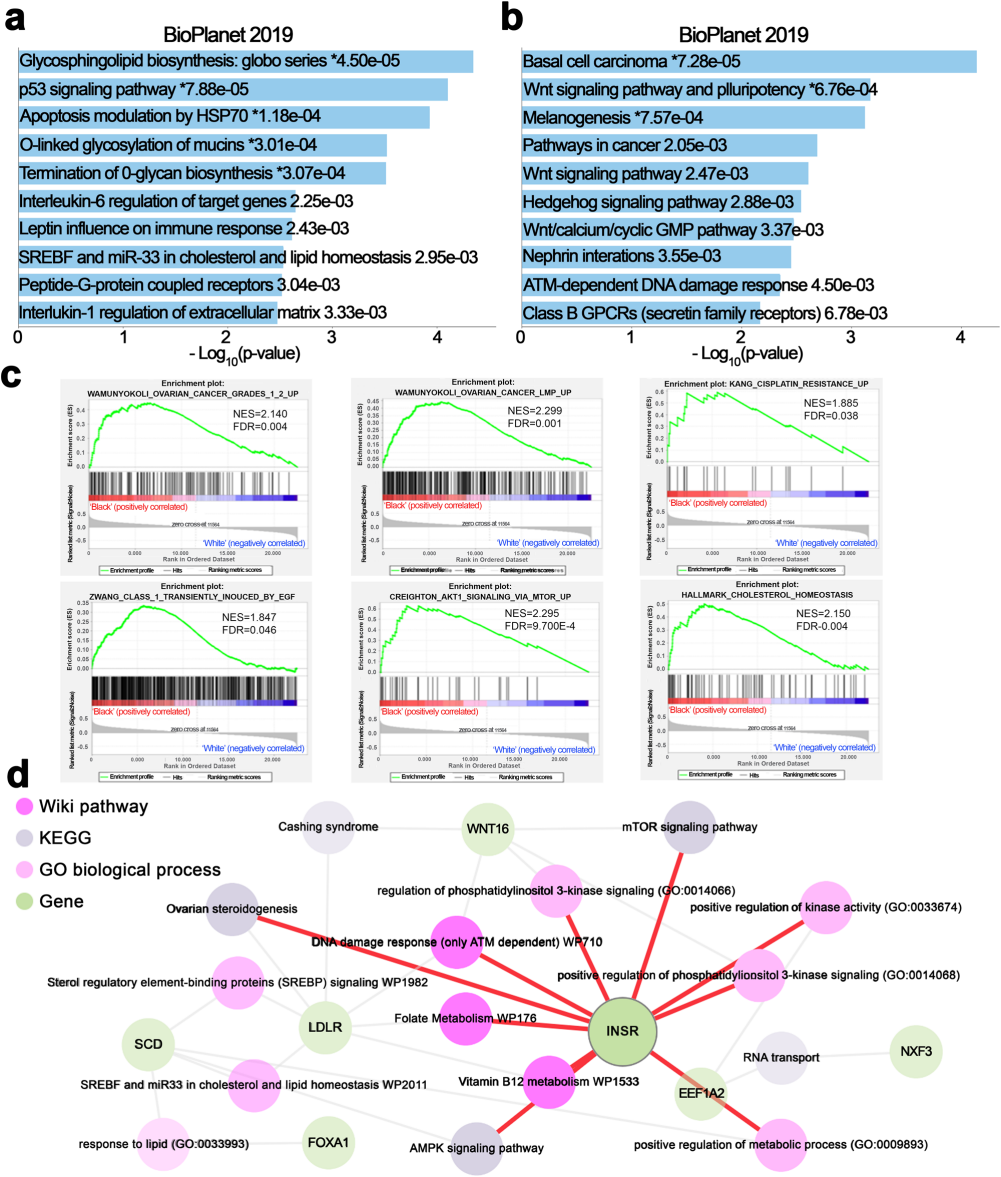
Integrative analysis of pathway enrichment and differential gene expression in ovarian tumors from Black vs White patients. **a**, **b** Pathway enrichment analysis based on the BioPlanet 2019 dataset, using the open website tool Enrichr (https://maayanlab.cloud/enrichr/), was performed with significantly up-regulated (**a**) or down-regulated genes (**b**) in Black vs. White ovarian cancer patients. The results are shown in a bar chart displaying significant biological processes and their corresponding p-values. **c** GSEA shows representative gene sets related to ovarian cancer disease, cisplatin resistance, and cell growth or lipid metabolism that are significantly enriched (FDR < 0.05) in the Black vs. White cohort. (NES, normalized enrichment score.) **d** The pathway interaction network provides a visualization of interconnected biological pathways derived from multiple sources, illustrating the connectivity between differentially expressed genes and enriched pathways using the open website tool Enrichr Knowledge Graph (Enrichr-KG) (https://maayanlab.cloud/enrichr-kg). Different shapes in the network correspond to the database of origin for each pathway, as indicated in the legend: Wiki Pathways 2021 human, KEGG 2021 human, GO Biological Process human, and Differential Genes.

To elucidate the underlying molecular mechanisms associated with the distinct transcriptomic profiles of tumors from Black and White patients, we used pathway enrichment and gene-set-enrichment analyses (GSEA). Pathway enrichment analysis using the upregulated genes in the BioPlanet 2019 database revealed “*glycosphingolipid biosynthesis”* (*p* = 4.50e-05), “*p53 signaling”* (*p* = 7.88e-05), and “*apoptosis modulation by HSP70”* (*p* = 1.18e-04) as the top 3 significantly enriched pathway (Fig. 2a), suggesting changes in cellular stress responses and apoptosis in tumors from Black patients. By contrast, the pathways enriched with down-regulated genes in tumors from Black patients, using the BioPlanet 2019 database, were mostly associated with “*WNT signaling”* (Fig. 2b). The Wnt pathway is implicated in processes critical to cancer progression, such as cell proliferation, apoptosis, immune response, resistance, and EMT/migration/invasion/metastasis, among others^13^.

GSEA revealed positive enrichment for “*ovarian cancer”* (NES = 2.140, FDR < 0.004) and “*drug resistance*” pathways (NES = 1.885, FDR = 0.038), in tumors from Black patients (Fig. 2c). Furthermore, GSEA indicated a positive enrichment for pathways “*transiently induced by EGF”* (NES = 1.847, FDR = 0.046), “*AKT signaling via MTOR”* (NES = 2.295, FDR = 9.700E-4), and “*cholesterol homeostasis”* (NES = 2.150, FDR = 0.04), highlighting that oncogenic and metabolism-related signals are enriched in tumors from Black patients.

An integrative analysis of DEGs using the WikiPathways Human 2021, KEGG Human 2021, and Human GO Biological Process databases revealed a complex interplay of genes and biological processes, including lipid metabolism mediated by SREBP and miR33 (genes represented *LDLR* and *SCD*), insulin signaling (gene *INSR*), and vitamin B12 metabolism (gene *EEF1A2*) (Fig. 2d). Key genes such as *FOXA1, LDLR*, and *WNT16* were also linked to these processes. Among them, INSR appears as a central gene interconnected with other signaling pathways (Fig. 2d). Collectively, our data support that oncogenic signals enriched in tumors from Black women may be driving disparities in outcomes.

### Validation of differentially expressed genes in tumors from Black vs White patients

**Fig. 3:**
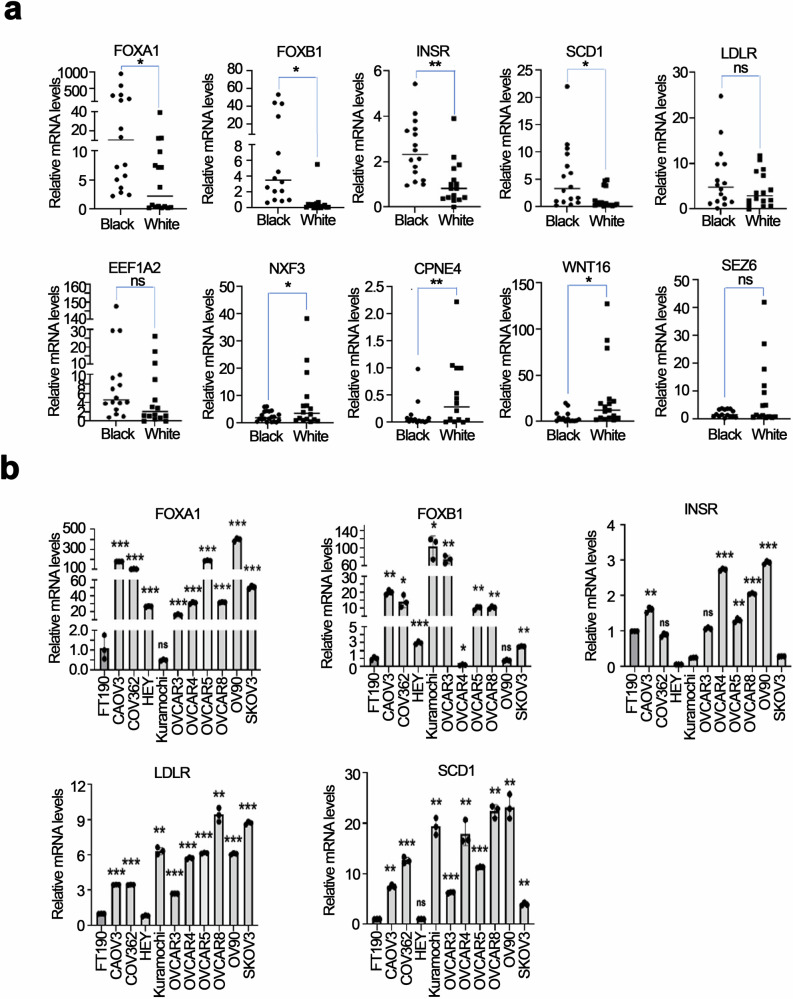
Validation of expression of DEGs in ovarian tumors and in OC cell lines. **a** Comparative analysis of mRNA levels in Black and White groups: Scatter plots demonstrate the relative mRNA levels of specific genes (*FOXA1, FOXB1, EEF1A2, LDLR, SCD1, INSR, NXF3, CPNE4*, and *WNT16*) between the Black and White groups as determined by qRT-PCR. 16 samples for each group randomly chosen based on nucleic acid availability. The error bars are presented as mean ± standard deviation (SD), with *n* = 3. * p < 0.05; ** *p* < 0.01; ns, not significant (*p* > 0.05). **b** qRT-PCR analysis showed mRNA expression levels of *FOXA1, FOXB1, LDLR, INSR*, and *SCD1* in various OC cell lines compared to levels in FT190 cells. The error bars represent the mean ± SD, with *n* = 3, * *p* < 0.05; ** *p* < 0.01; *** *p* < 0.001; ns, not significant (*p* > 0.05).

Quantitative RT-PCR (qRT-PCR) was performed using RNA extracted from tumors in this cohort to validate the expression levels of selected DEGs identified through RNA-seq analysis. The *mRNA* levels of *FOXA1, FOXB1*, *SCD1* and *INSR* were significantly higher (*p* < 0.05) in tumors from Black compared to White patients, while the *mRNA* levels of *NXF3, CPNE4*, and *WNT16* were significantly lower in tumors from Black patients (Fig. 3a). The *mRNA* levels of *EEF1A2* and *LDLR* detected as being differentially expressed between tumors from Black vs. White patients through RNA-seq, were not found to be significantly different by qRT-PCR, although there was a trend towards upregulated expression in tumors from Black women (Fig. 3a).

There are no available preclinical models to study potential biological race-based differences in OC. Given limited information regarding the function of these proteins in OC and to determine their potential involvement in cancer, we compared the *mRNA* levels of these genes in OC cell lines with FT190, immortalized normal fallopian tube cells. *FOXA1, FOXB1, LDLR, SCD1*, and *INSR* levels were higher in most cancer cell lines (Fig. 3b). Western blot (WB) analysis assessed protein expression levels of *INSR* and *FOXA1* across various OC cell lines compared to the non-transformed cell line FT190. Both INSR and FOXA1 were expressed at higher levels in OC cell lines than in FT190 cells, supporting a potential association with cancer (Supplementary Fig. 2). However, the expression of these proteins varied among cell lines, with the highest INSR expression observed in OVCAR4 and OV90 cells, and the highest *FOXA1* expression in OVCAR5 and OV90 cells. Conversely, INSR expression levels were lower in SKOV3 cells; while *FOXA1* expression levels were not detected in OVCAR3 and SKOV3 cells (Supplementary Fig. 2). Taken together, these analyses show that FOXA1 and INSR are variably expressed in OC models.

### Function of Insulin-INSR signaling axis in OC models

**Fig. 4:**
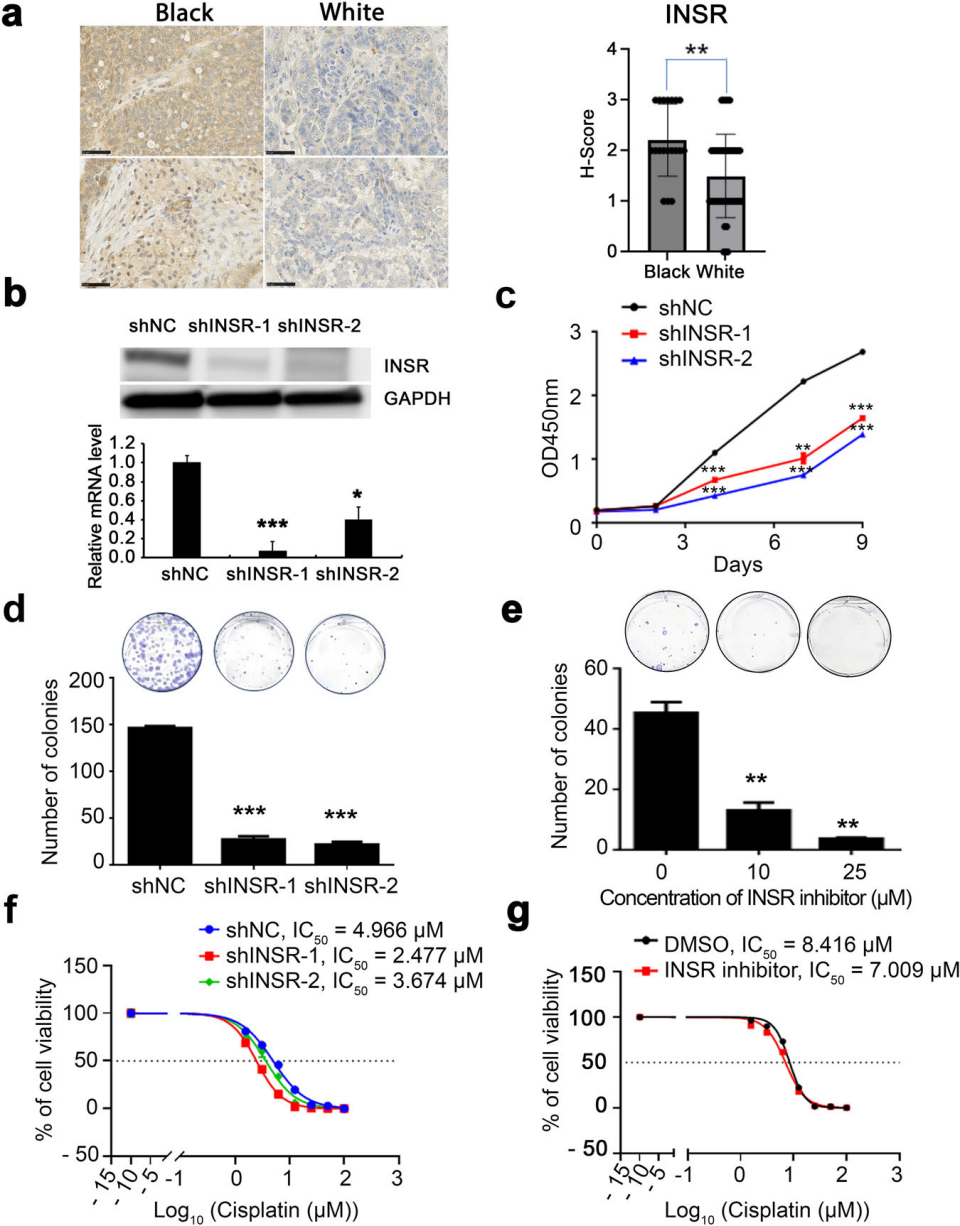
INSR expression and function in ovarian cancer cells. **a** Representative micrographs (left) and H-Scores (right, the error bars represent the mean ± SD. **p < 0.01) of insulin receptor IHC staining performed using a human tissue microarray (TMA) that included ovarian cancer specimens from 10 Black and 22 White individuals. **b** Quantification of INSR protein levels by Western immunoblotting in OV90 cells transfected with INSR shRNAs (shINSR-1 and shINSR-2), or scrambled shRNAs (shNC) and mRNA expression levels by qRT-PCR (bottom, the error bars represent mean ± SD, *p < 0.05; ***p < 0.001). **c** Cell proliferation curves measured using the CCK8 assay of OV90 cells stably transduced with shNC, shINSR-1, or shINSR-2. The error bars represent the mean ± SD. ** *p* < 0.01, ***p < 0.001. **d** Clonogenicity measured with a colony formation assay (CFA) of OV90 cells transfected with shNC (Control), shINSR-1, and shINSR-2. The upper panel image displays representative colonies, and the lower panel shows numbers of colonies (Bar graph illustrates mean numbers of colonies ± SEM, with *n* = 3, ***p < 0.001). **e** CFA measures the effect of an INSR inhibitor on the colony-forming potential of OV90 cells (Bar graph illustrates mean numbers of colonies ± SEM, with *n* = 3, *p < 0.05). **f** A cell viability CCK8 assay shows responses expressed as IC_50_ values of OV90 cells transduced with shNC, shINSR-1, and shINSR-2 to cisplatin. **g** Effects of cisplatin on cell viability (CCK8 assay) of OV90 cells treated with DMSO or an INSR inhibitor (25 µM). The cisplatin IC_50_ values for each treatment are indicated.

Having identified INSR as highly expressed in tumors from Black women and knowing that this receptor plays an oncogenic role in several cancers^14,15^, we next focused on its potential functions in HGSOC. Insulin binds to the INSR, a receptor tyrosine kinase (RTK), which in turn activates intracellular signaling through insulin receptor substrate (IRS) 1 and 2 proteins and downstream stimulation of PI3K and ERK^16^. In cancer, insulin signaling is a major factor linking obesity to cancer progression and has been implicated in resistance to chemotherapy^17^. We confirmed differential INSR expression in HGSOC through immunohistochemical (IHC) analysis using a tissue microarray (TMA) comprising 10 tumors from Black and 22 tumors from White women, a separate cohort from the specimens analyzed through RNA-Seq. Patients’ characteristics are in Supplementary Table 6. The H-score, calculated based on staining intensity and the percentage of positive cancer cells, was determined in a blinded manner by a board-certified pathologist. INSR expression, typically membranous or cytoplasmic, was higher in tumors from Black women compared to those from White women (Fig. 4a, *p* = 0.002). To elucidate the receptor’s role in HGSOC models, we used genetic and pharmacological methods to deplete or inhibit INSR. Transduction of short hairpin RNA (shRNA) led to knock down of INSR in OV90 cells at both *mRNA* and protein levels compared to scrambled shRNA (Fig. 4b). Downregulation of INSR markedly inhibited the proliferation rate (Fig. 4c) and colony-forming ability (Fig. 4d) of OV90 cells, supporting an oncogenic function of this receptor. We next determined the effects of an INSR inhibitor, HNMPA-(AM)3, in OV90 cells. Consistent with the effects of INSR knockdown, the inhibitor decreased OVCAR5 cell proliferation and the colony formation ability of OV90 and OVCAR5 cells (Fig. 4e, Supplementary Fig. 3a, b). Given that the insulin-INSR signaling axis mediates ERK phosphorylation, OC cells were stimulated with insulin in the presence of control (PBS) or of the INSR inhibitor. HNMPA-(AM)3 decreased ERK phosphorylation stimulated by insulin-(AM)3 (10 μM and 25 μM; Supplementary Fig. 3c, d).

As resistance to platinum-based treatment is a key determinant of clinical outcome in OC and is more frequently observed in Black compared to White women^18^, we assessed the effects of INSR on responsiveness to cisplatin. INSR knockdown increased OC cells’ sensitivity to cisplatin as shown through decreased IC_50_ values (shNC: IC50 = 4.966 µM; shINSR-1: IC50 = 2.477 µM; shINSR-2: IC50 = 3.674 µM; Fig. 4f). Similarly, OV90 cells treated with an INSR inhibitor also showed modest increased sensitivity to cisplatin (DMSO: IC50 = 8.416 µM; INSR inhibitor: IC50 = 7.009 µM; Fig. 4g), supporting the potential role of INSR as a survival factor in OC cells. In parallel, we assessed the effects of insulin, which is the INSR ligand. Modest changes in sensitivity to cisplatin were observed in OVCAR4 and OVCAR5 cells treated with insulin (Supplementary Fig. 4a, b). Insulin-treated OV90 and OVCAR5 cells had an increased proliferation rate (Supplementary Figs 4c and d) and colony-forming ability (Supplementary Fig. 4e), suggesting a growth-promoting effect of insulin in this context. Collectively, these results point to oncogenic functions of INSR, which is highly expressed in tumors from Black women, supporting its potential role as a determinant of more aggressive OC phenotype.

### Function of FOXA1 in OC models

**Fig. 5:**
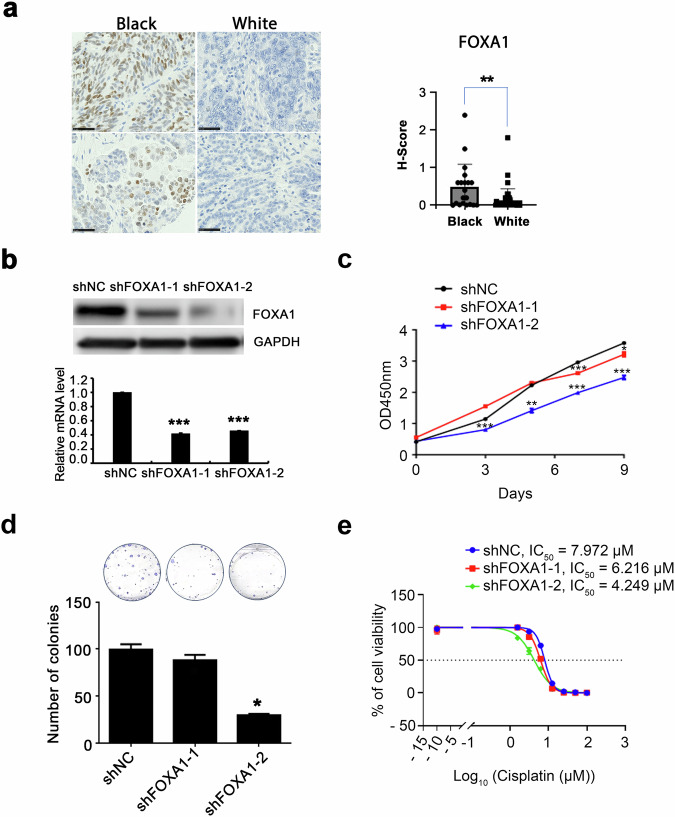
FOXA1 modulates cell proliferation and sensitivity to cisplatin in ovarian cancer cells. **a** Protein levels of FOXA1 determined by IHC in sections of ovarian cancer tumors from Black (*n* = 10) and White (*n* = 22) patients. Left panel, representative pictures of IHC staining; right panel, evaluation of staining intensity (H-scores, The error bars are defined as mean ± SD, **p < 0.01). **b** FOXA1 protein levels by Western blot (top) and mRNA measured (qRT-PCR, bottom) in OVCAR5 cells stably transduced with shNC (non-targeting control) or one of two shRNAs (shFOXA1-1 or shFOXA1-2) targeting FOXA1. The error bars represent the mean ± SD. (***p < 0.001, *n* = 3). **c** Proliferation of OVCAR5 cells following stable transduction with shNC, shFOXA1-1, or shFOXA1-2. The error bars are defined as mean ± SD, with *n* = 3, *p < 0.05, **p < 0.01, ***p < 0.001. **d** CFA measures the number of colonies formed by OVCAR5 cells following transduction with shNC, shFOXA1-1, or shFOXA1-2. Top, representative pictures of colonies; bottom, quantification of colony numbers (Bar graph illustrates mean numbers of colonies ± SEM, with *n* = 3, *p < 0.05). **e** Cell viability assay after culturing shNC, shFOXA1-1, or shFOXA1-2 transduced OVCAR5 cells with increasing concentrations of cisplatin (IC50 values) of to.

FOXA1, found among the top upregulated genes in tumors from Black women (Supplementary Table 4), is a pioneer factor that regulates the transcriptional activity of nuclear receptors modulating estrogen or androgen signaling in hormone receptor-positive breast cancer^19,20^ and in prostate cancer^21^ and its upregulation was shown to contribute to resistance to hormone receptor blockade^22^. In addition to its transcriptional pioneering function, FOXA1 was recently shown to inhibit interferon signaling in breast and prostate cancer models^23^. These functions suggest the possibility that FOXA1 could be contributing to immune evasion and to an aggressive phenotype in Black patients. Validation studies used IHC analysis of a separate cohort of HGSOC tumors from Black and White patients. FOXA1 was expressed at higher levels, as measured by H-scores in tumors from Black vs. White patients, with predominantly nuclear staining observed (Fig. 5a). To begin to investigate its functions, FOXA1 knockdown (KD) cells were generated from OVCAR5 cells by using lentiviral transduction with shRNAs targeting FOXA1 (shFOXA1-1 and shFOXA1-2). Successful knockdown of FOXA1 at the mRNA and protein levels was demonstrated by qRT-PCR and western blotting (Fig. 5b). FOXA1 knock down inhibited cell proliferation compared to shNC control cells (Fig. 5c) and colony formation assay vs control group (Fig. 5d). Furthermore, FOXA1 KD increased sensitivity to cisplatin compared to the control group, as evidenced by lower IC50 values (Fig. 5e). The increased sensitivity to cisplatin suggested a possible protective role of FOXA1 in OC cells. Our results identify new roles of FOXA1 in regulating both the proliferative capacity and drug sensitivity in OC cells.

### Immunological differences between Black and White cohorts

**Fig. 6:**
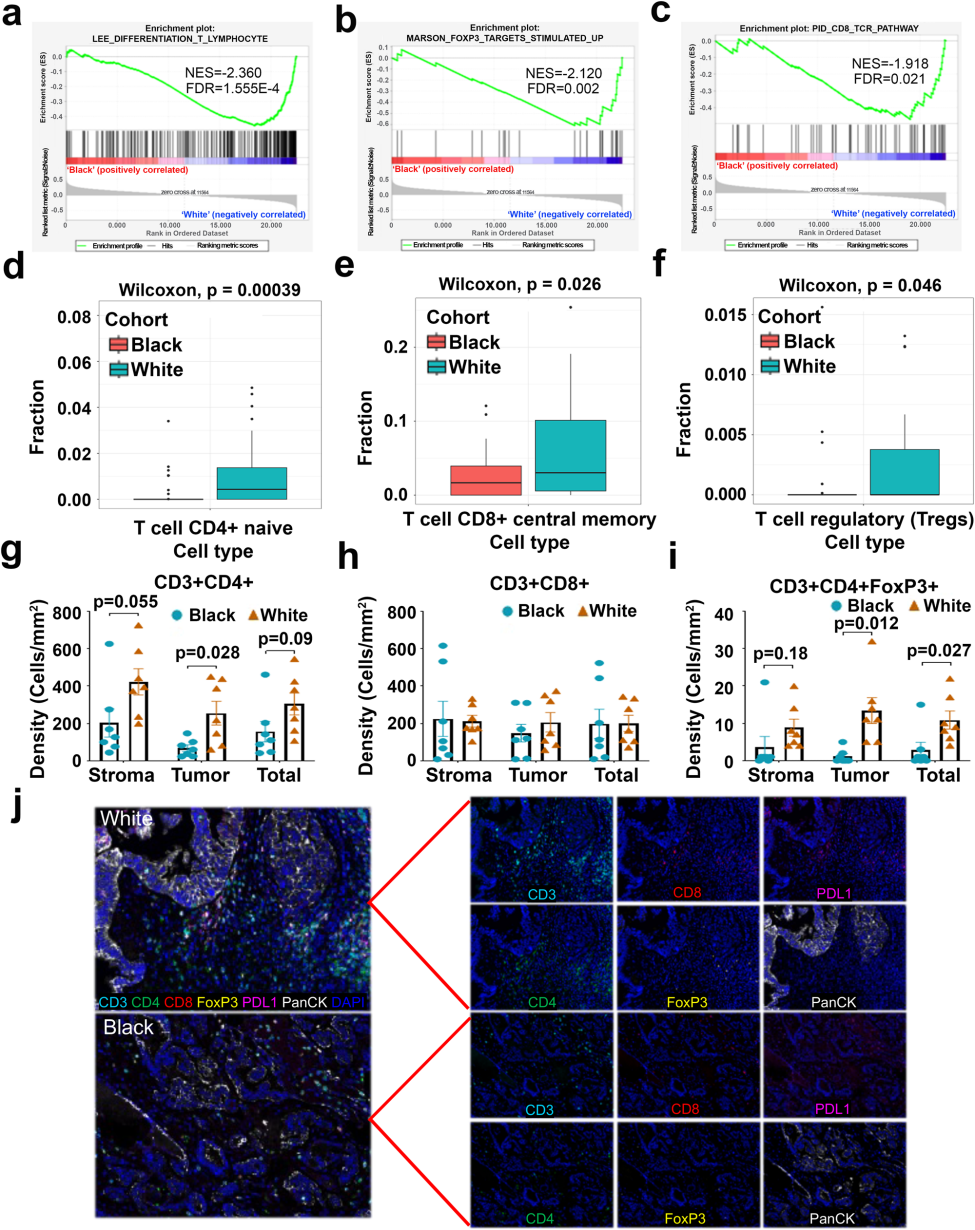
Comparative immune profiling of T cell subpopulations in tumors from Black and White cohorts. **a**–**c** GSEA Enrichment plots based on gene expression levels (RNAseq) in the Black vs. the White cohort for CD8 T-lymphocyte differentiation (a), genes upregulated in memory T-cells after Marson’s stimulation (**b**), and genes associated with CD8 T-cell memory (**c**). **d**–**f** Comparative analysis of immune cell populations using gene expression data (RNAseq) and deconvolution techniques by using the X-cell algorithm in Black and White patients. Bar graphs (The error bars are defined as mean ± SD, Wilcoxon test) show the proportions of CD4+ naive cells (**d**), and CD8+ central memory cells (**e**), regulatory T cells (Tregs) (**f**). **g**–**i** Immune cell infiltration measured by multiplex IHC in stromal and tumor tissues between Black and White ovarian cancer patients. Bar graphs (The error bars are defined as mean ± SE, with *n* = 15 and 17, respectively.) illustrate the densities of CD3 + CD4 + T cells (**g**), CD3 + CD8 + T cells (**h**), and CD3 + CD4 + FoXP3+ T cells (**i**) in stromal, tumor, and total cell populations. **j** Representative mIHC staining images of tumors from White and Black patients.

As the immune system plays a key role in harnessing cancer progression and differences in immune characteristics have been reported between Black and White populations in other cancer types^24,25^, we evaluated potential differences in HGSOC. GSEA plots extracted from the RNA-seq analysis pointed to significant differences in the activity of immune-related gene sets (“*T-Lymphocytes”; “FOXP3 targets”*, and *“CD8 TCR pathway*”) between Black and White cohorts, which could reflect variation in immune system functions (Fig. 6a–c). The reduced activity of these pathways in tumors from Black patients suggest attenuated anti-tumor immune response, which could correlate with a more aggressive disease course and decreased survival.

Next, immune cell-type abundance estimates based on RNA-seq results were performed using deconvolution techniques with X-cell^26^. Five immune cell-types were predicted to be enriched in tumors from White patients, including B-cells (Class-switched memory B cells; Supplementary Fig. 5a) and T-cells: CD4+ effector memory (Supplementary Fig. 5b), CD4+ naive, CD8+ central memory, and regulatory T-cells (Fig. 6d–f). The difference in the overall fraction of B cells tended to be lower but did not reach statistical significance in tumors from Black vs White patients (*p* = 0.078; Supplementary Fig. 5c). These findings suggest that differences in the immune composition of HGSOC tumors may exist, with potential implications for tumor progression and treatment outcomes.

Immune cell infiltration was validated using multiplex color immunohistochemistry (mIHC) in a separate group of tumor specimens from Black (*n* = 15) and White (*n* = 17) patients. The density of specific immune cell types was assessed in different tumor regions (stroma and tumor). There was a significantly lower density of CD3 + CD4 + T cells in the tumors of specimens from Black patients, with a trend towards decreased density in the stroma (Fig. 6g, j). No change in the infiltration of CD8 + T cells was observed in tumors or stroma between specimens from Black and White patients (Fig. 6h, j). Tumor specimens from Black patients displayed a significantly lower count of T regulatory cells across the tumor and a trend toward lower counts in the stroma and total area compared to tumors from White patients (Fig. 6i, j), consistent with observations from RNA-seq analysis. Additionally, the number of PDL1 staining cells in tumor, stroma and all area, as well as PDL1 intensity on tumor cells was significantly lower in tumors from Black vs. White women (Supplementary Fig. 5d, e). These results, albeit in a limited cohort, show differences in the immune microenvironment of tumors from Black vs. White women.

## Discussion

While social vulnerabilities play an important role in race-based treatment outcomes, biological factors governing disease course and response to treatment may also contribute to the observed differences in clinical outcomes. These biological factors may be influenced by social determinants of health, including stress and physiological effects associated with the experience of racism which have been shown to influence disease course and mortality in other contexts^27–29^. Here, we conducted the first large scale methylomic and transcriptomic analysis of HGSOC tumors from Black and White women with the goal of identifying biological differences in tumors and potential drivers of disparity. Methylation profiling identified several chromosomal regions and several promoters associated CpG sites being differentially methylated while transcriptomic analyses identified >200 DEGs associated with pathways related to metabolism and immune responses in tumors from Black vs White women. We focused functional studies on a few key genes with increased expression levels in tumors from Black women. We found INSR and FOXA1 to be upregulated in tumors from Black women and hypothesize that they may play a role in the more aggressive biology of OC in Black women and diminished response to platinum-based therapy. Our immunological analyses, albeit limited, also point to potential race-based differences in immune cell infiltration, suggesting a less robust anti-tumor immune response in tumors from Black patients. Our findings have several important implications.

First, despite the recognition of race-based disparities in health outcomes in OC^30^, the understanding of how race-based biological factors affect OC disparities remains limited. There also remains a lack of suitable research models for studying these differences, further impeding progress in understanding distinct biological drivers. A recent genome-wide association study found ten distinct single nucleotide polymorphisms (SNPs) associated with the development of OC in women of African American ancestry, including SNPs for AKR1C3, follistatin (FST), MAGEC1, and *GK2*, which had not been identified in European populations^31^. Another study did not identify significant differences in the prevalence of germline BRCA 1 and 2 mutations in tumors from Black patients^32^. Similarly, in our cohort, the number of known BRCA mutated tumors was similar among the Black and White patients. No large-scale studies have examined transcriptomic or epigenomic signature differences in HGSOC tumors from Black vs White women.

Here we identified 277 differentially expressed transcripts between tumors from Black vs White patients. Among the differentially expressed genes, k-means clustering (k = 3) displayed three distinct and well-defined clusters of genes between the two cohorts. One particularly notable cluster included genes predominantly upregulated in specimens from Black patients. It contained multiple genes involved in regulation of transcription: *KBTBD11, FOXA1, FOXB1, RRN3*. This cluster also included multiple genes encoding for proteins related to metabolic signaling: *C2CD4C*, *INSR*, and *SLC7A2. C2CD4C* belongs to a family encoding for proteins with regulatory roles for insulin secretion^33^. Some of the identified DEGs were involved in lipid and cholesterol pathways, ovarian cancer-related oncogenic and drug resistance pathways, such as Wnt signaling, p53 apoptosis, and the DNA damage pathway. Notably, lipid metabolism reprogramming, and increased cholesterol content were recently associated with platinum resistance, by us and other groups^34,35^. Our findings should be interpreted with caution, as the tumor cohort is small and information about clinical co-variates is limited.

Second, among the DEGs, we identify *INSR* as being highly upregulated in tumors from Black patients. INSR is upregulated in other epithelial malignancies where it promotes tumor growth^15^. The functions of the receptor have not been elucidated in OC. Still, polymorphisms in the INSR gene have been linked to increased risk for OC and with sensitivity to platinum-based chemotherapy^36,37^ supporting a potential role. Here we show that the insulin-INSR axis drives OC cell proliferation and influences response to platinum, supporting the idea that overexpression of INSR could drive tumor progression. Interestingly, insulin signaling is a major factor linking obesity to cancer progression and has been implicated in resistance to chemotherapy^17^. Visceral and childhood obesity has been associated with an increased risk of OC, particularly in the pre-menopausal period^38,39^. This association is particularly significant in Black women, in whom increased body mass index and weight gain after age 18 strongly correlates with OC incidence^40^. Obesity is also associated with insulin resistance^17^ and high insulin levels, and women with high insulin levels carry an increased risk of OC^41^. A trend to higher BMI was also observed among Black patients in our cohort. Thus, our findings regarding INSR upregulation in tumors from Black women could link a more aggressive tumor phenotype to the metabolic syndrome commonly diagnosed in Black patients.

Third, we identified multiple T-cell-related pathways being enriched in the gene expression data from the White patient group. Cell-type deconvolution analysis demonstrated the enrichment of specific immune cell types within this cohort, including several T-cell subpopulations and class-switched memory B cells. The decreased frequency of memory T and B cells in Black patients could impact the robustness of the immune response, leading to weaker immune surveillance against cancer. Validation by multiplex IHC and cell density estimation confirmed enrichment in T-helper (CD3 + CD4 + ) in tumors from White patients. Surprisingly, T-regulatory cells (CD3 + CD4+FoxP3 + ) which have a suppressive role were also detected in higher frequency in White women.

Race-based differences in anti-tumor immune response have been described in other tumor types with various and sometimes contradictory observations. An increased frequency of plasma cells and IgG secretion was reported in prostate cancer tumors from Black men, supporting a more active B cell response in this context^42^. Likewise, triple-negative breast cancer tumors from Black women were infiltrated by increased numbers of CD45+ lymphocytes compared to tumors from White patients^43^. Another study confirmed that breast tumors from Black patients were infiltrated by increased numbers of immune cells but found that increased numbers of exhausted CD8 + T cells^44^ characterized the immune milieu. In contrast, a cold tumor microenvironment devoid of vasculature and infiltrating immune cells was reported for lung tumors from Black compared to White patients^45^. Racial disparities in breast and prostate cancer have been attributed to chronic or recurring inflammation driven by mechanisms of innate immunity. Variability in the expression of genes related to innate immunity due to SNPs that are specific to populations based on race has been reported^46^. Immune cell infiltration was recently also assessed in HGSOC tumors from Black and White patients, with no obvious differences in immune cell percentages between the cohorts^47^. While the numbers of tumor infiltrating lymphocytes (TILs) and CD8 + T cells correlated with clinical outcomes in all patients and in White patients, in this study, this association was attenuated among Black patients^47^. However, the abundance of TILs, CD8 + T cells and the spatial clustering of immune cells correlated with survival in a larger cohort from the African American Cancer Epidemiology Study^48^.

Interestingly, we observed a lower intensity of PD-L1 staining and lower number of PD-L1 staining cells in tumors from Black patients. Recent studies reported that increased expression of PD-L1 on immune cells in the tumor milieu, including on tumor-associated macrophages, was associated with increased total numbers of infiltrating lymphocytes and with better survival in HGSOC^49,50^. PD-L1 expression was also correlated with increased lymphocyte infiltration in a cohort of Black women with HGSOC^51^. The observed differential PD-L1 expression between Black and White patients could reflect impaired anti-tumor immunity and/or impact responsiveness to immune interventions and should be validated in a larger tumor set.

In all, our results identify distinct drivers linked to metabolic and oncogenic pathways in HGSOC tumors from Black women that might account for more aggressive biology and decreased response to platinum-based treatment. Immuno-phenotyping of a subset of tumors identified lower PD-L1 intensity and decreased T-cell engagement in tumors from Black women which may also contribute to accelerated tumor progression and poorer clinical outcomes. These results should be validated in a larger tumor cohort and correlated with clinical co-variates and biomarkers of stress response. Continued efforts to decipher biological determinants of health care disparities in HGSOC will put forward potential new strategies to tackle the aggressive disease pattern of HGSOC in Black women.

## Methods

### Tumor specimens

In this manuscript we use ‘Black’ to refer to non-Hispanic Black or African American individuals and ‘White’ to refer to non-Hispanic White (NHW) individuals. Frozen HGSOC tumors from Black (*n* = 35) and White (*n* = 31) consenting patients were collected from the biorepositories of four academic medical centers, Northwestern Medicine or partner institutions (University of Chicago, Indiana University, University of North Carolina) and the biorepository of the NRG Oncology. Nucleic acids extracted from these specimens were used for RNA-Seq, methylation analyses and RT-PCR verification. The race of all participants was self-reported. Eligibility for the study included high grade serous histology and stage III/IV ovarian cancer. All patients were women, age was not an exclusion factor. Patients’ characteristics are in Supplementary Table 1. A tissue microarray (TMA) was constructed in the Northwestern University Pathology Core using HGSOC paraffin-embedded tumor cores from 10 Black and 22 White patients; each tumor being represented in duplicate. Patients’ characteristics are in Supplementary Table 6. Additional paraffin-embedded HGSOC tumor slides from 15 Black and 17 White patients were obtained from the biorepository of Northwestern University and the University of North Carolina. All patients provided written informed consent to tissue banking and use of tissue for research. All studies involving human participants or samples were conducted in accordance with relevant ethical regulations, including the Declaration of Helsinki, and were approved by the appropriate institutional review board (IRB) (STU00212683).

### Cell culture

OVCAR5 cells were a gift from Dr. Marcus Peter, Northwestern University; OVCAR4 cells were from Dr. Mazhar Adli, Northwestern University; immortalized human fallopian tube luminal epithelial cells (FT190) were from Dr. R. Drapkin of University of Pennsylvania; OV90 cells were purchased from the American Type Culture Collection (ATCC). Cells were maintained in a 37 °C incubator with 5% CO_2._ Low passage cells were used, and all cell lines were authenticated (IDEXX BioAnalytics) and tested to be pathogen and Mycoplasma negative (Charles River Research Animal Diagnostic Services). OVCAR5 cells were maintained in RPMI-1640 with L-glutamine (Corning, Cat# 10-040-CV) plus 10% FBS, 1% GlutaMAX (Gibco, Cat# 35-050-061), and 1% penicillin-streptomycin. OV90 cells were cultured in a medium comprising a 1:1 mixture of MCDB 105 (Sigma Aldrich, Cat# M6395) and Medium 199 (Corning, Cat# 10-060-CV), supplemented with 15% FBS. All cell culture media were supplemented with 10% FBS (Fisher Scientific, Cat# 35011CV) and 1% penicillin-streptomycin solution (Corning, Cat# 30-002-CI).

### Chemicals and reagents

INSR inhibitor HNMPA-(AM)3 (CAS 120944-03-8) (cat. no. sc-221730) was purchased from Santa Cruz. Insulin (cat. no. I0516), Cisplatin (CAS 15663-27-1) (cat.no. 232120), and dimethyl sulphoxide (DMSO, cat. no. D2650) were from Sigma-Aldrich.

### gDNA and RNA extraction

Genomic DNA (gDNA) was extracted from human tumor tissues using the DNeasy Blood & Tissue Kit (Qiagen, Valencia, CA, Cat. # 69504). Total RNA was extracted using a RNeasy Mini kit with on-column DNA digestion (Qiagen, Cat. # 74104). The concentrations of gDNA and RNA were measured with the NanoDrop™ 2000 and Qubit spectrophotometry. Purity was estimated by calculating the absorbance ratio at 260/280 nm. DNA and RNA were used for RNA sequencing and methylation profiling, respectively.

### Infinium Methyation EPIC array data processing

gDNA (500 ng) was bisulfite converted and used for DNA methylation profiling at the NUSeq Core Facility, Northwestern University, using Epic methylation arrays according to the Illumina’s protocol. BeadChips were scanned with an Illumina iScan and then analyzed using the Illumina GenomeStudio software. Methylation array data were collected in *idat* format. Nine multi-aliquot samples existed, for which the mean beta values were used to represent each sample. After aliquot averaging, 35 black patient samples and 31 white patient samples remained for analysis. R package SeSAMe^52^ was used to process IDAT files that were generated from EPIC array and downstream differential methylation locus (DML) and region (DMR) analysis. “openSesame” from SeSAMe was used to process IDATs to DNA methylation level (aka β value) matrices in R. In DML, SeSAMe utilizes linear models to identify DMLs between two cohort groups, white and black, from DNA methylation values. In DMR, neighboring CpGs that show consistent methylation variation were merged into differentially methylated regions (DMRs), and adjusted pvalue was calculated using Benjamini-Hochberg procedure.

### RNA-sequencing (RNA-seq) and data processing

RNA-seq data was collected as *fastq* read files and aligned using STAR. Expected read counts were estimated using RSEM and filtered for low expression. Three patient samples were removed during outlier detection, after inspection by principal component analysis. After quality control, 35 Black patient samples and 28 White patient samples remained for downstream analysis. RSEM expected counts were rounded to the nearest integer and used for downstream differential analysis. Normalization of expected counts was performed using DESeq2. After filtering, all downstream analysis was performed on 22,511 gene-features across *n* = 63 total samples.

### Survival analysis

Clinical information (survival) for ovarian cancer patients was downloaded from the cbioportal website (https://www.cbioportal.org/) for HGSOC^12^, Ovarian Serous Cystadenocarcinoma (TCGA, Firehose Legacy) and Ovarian Serous Cystadenocarcinoma (TCGA PanCancer Atlas)^13^. Duplicated patient IDs were removed, resulting in a total of 1230 patients. Among those patients, 531 patients were self-identified as White and 37 as Black based on “Race Category” and were included in this analysis. Survival analysis was performed by using the Kaplan-Meier plot method for Black and White patients.

### Quantitative real-time RT-PCR (qRT-PCR) analysis

QRT-PCR was used for validation. Total RNA (1 μg) was reverse transcribed into cDNA by using the Applied Biosystems™ High-Capacity cDNA Reverse Transcription Kit (Applied Biosystems) according to the manufacturer’s instructions. Quantitative real-time PCR analysis was performed by using Applied Biosystems™ Power SYBR™ Green PCR Master Mix (Applied Biosystems) and an AB 7900HT instrument (Applied Biosystems, Foster City, CA). 18S RNA was used as endogenous control. Sequences of primers for *FOXA1, FOXAB1, EEF1A2, LDLR, SCD1, INSR, NXF3, CPNE4, WNT16* are included in Supplementary Table 7. The RT-PCR reaction used the following parameters: 94 °C for 10 min, followed by 40 cycles of amplification at 94 °C for 15 s and 60 °C for 1 min, and an extension step of 7 min at 72 °C. The relative expression of target genes was calculated by using the ΔΔCt method. Results are presented as means ±SD of replicates. Measurements were performed in triplicate for each sample.

### Western blotting (WB)

Cells were lysed using the radio immunoprecipitation assay (RIPA) buffer. After sonication and centrifugation, protein concentrations were quantified by using the Bradford assay (Bio-Rad Protein Assay Reagent, Bio-Rad, Berkeley, CA). Equal amounts of protein were resolved by SDS-PAGE and transferred to PVDF membranes. After blocking in 5% non-fat milk, the membranes were incubated with primary antibody overnight at 4°C and then with secondary antibody. Detection used the SuperSignal West Pico PLUS Chemiluminescent Substrate (Thermo Scientific). Anti-INSR and anti-FOXA1 antibodies were purchased from Abcam (Cambridge, Massachusetts; Cat # ab137747 and ab170933, respectively).

### Immunohistochemistry (IHC)

IHC was performed in the Lurie Cancer Center Pathology Core. The TMA and other paraffin embedded tumor slides were deparaffinized using xylene. Sections were rehydrated with decreasing ethanol concentration and incubated in epitope unmasking buffer (10 mM sodium citrate buffer; pH = 6) at 95 °C for 30 min. The sections were then blocked using 3% hydrogen peroxide for 15 min, followed by 3% normal goat serum (NGS), for 30 min. The sections were incubated with 1:250 anti-INSR antibody (ab137747; Abcam) and anti-FOXA1 [EPR10881] antibody (ab170933; Abcam) in 3% NGS overnight at 4 °C, after several dilutions had been tested. Negative control using serum only was used. The sections were then processed using LSAB2 Kits, Universal, HRP. Rabbit/Mouse kit (K0675; Agilent Technologies, Santa Clara, CA), followed by DAB + , liquid chromogen (K3467; Agilent Technologies, Santa Clara, CA), according to the manufacturer’s instructions. The sections were counterstained using hematoxylin (CS700; Agilent Technologies, Santa Clara, CA) and fixed using Faramount aqueous mounting medium (S302580-2; Agilent Technologies, Santa Clara, CA). A board-certified pathologist interpreted and scored the slides, blinded to the identity and race of patients. An H-Score was calculated based on the intensity (0-3 + ) and percentage of cancer cells staining (0-100%), using the formula: intensity x percentage/100.

### Gene Set Enrichment Analysis

Gene Set Enrichment Analysis (GSEA) used RNA-seq counts from 35 black patients and 28 white patients (63 patients in total) collected and processed as described previously^53^. Hallmark and C2 curated gene sets were selected for the analysis.

### Pathway enrichment analysis

Pathway enrichment analysis was performed by using Enrichr (https://maayanlab.cloud/enrichr/) In addition, functional analysis of genes was performed with Enrichr Knowledge Graph (Enrichr-KG), which combines enrichment analysis with a knowledge graph data representation to query a large collection of processed datasets comprising associations between genes and many biological and biomedical terms. This tool includes 26 gene set libraries from various categories, such as transcription, pathways, ontologies, diseases/drugs, and cell types. The study utilized three foundational pathway sets: Wiki 2021 Human, KEGG 2021 Human, and Go Biological Process 2021 Human, to analyze predicted top differential potential target genes. This approach facilitates the discovery of gene-gene links and reveals hidden relationships between genes and enriched terms from diverse datasets and resources. Enrichr-KG is an openly accessible tool, available at the provided URL (https://maayanlab.cloud/enrichr-kg)^54^.

### Gene knockdown

The INSR gene was knocked down in OV90 cells by transduction with lentiviral particles containing shRNAs (shINSR-1 or shINSR-2) targeting human INSR (MilliporeSigma, Cat#: CSTVRS). OV90 cells transduced with a scrambled shRNAs (shNC) (MilliporeSigma, Cat#: SHC003V) served as controls (shNC). Transfected cells were selected with puromycin. A similar procedure was used to knock down FOXA1 in OVCAR5 cells, which were transduced with lentiviral particles containing shRNAs targeting human FOXA1 including 2 sequences (shINSR-1 and shINSR-2) which were purchased from Origene Technologies (Rockville, MD, Cat#: TL312942V).

### Cell viability assay

Cells were treated with various concentrations of cisplatin for 24 h. Afterward, the medium was refreshed, and culture continued for 72 h. Cell viability was assessed using a Cell Counting Kit 8 (CCK8, Dojindo Molecular Technologies, Cat# CK04, Rockville, MD, USA), following the manufacturer’s protocol. Absorbances at 450 nm were recorded using a microplate reader (BioTek ELX800, BioTeK, Winooski, VT). The IC50 values were determined from these readings by using Prism (GraphPad software).

### Colony formation assay

Two hundred cells per well were seeded in triplicate per each experimental condition in 6-well-plates. The media was changed every three days. After 2 weeks of growth, cultures were washed with PBS, fixed with 70% ice-cold ethanol, and stained with 1% crystal violet. Colonies were counted in each well and average numbers per condition were compared.

### Cell proliferation assay

Cells were seeded in 96-well plates. Cell numbers were estimated by using a Cell Counting Kit 8 (CCK8, Dojindo Molecular Technologies, Cat# CK04, Rockville, MD, USA) at different time points, following the manufacturer’s protocol. Absorbances at 450 nm were recorded using a microplate reader (BioTek ELX800, BioTeK, Winooski, VT).

### Analysis of immune cell types

To examine differences in immune cell types between the Black and White patient groups deconvolution analysis was performed using the xCell algorithm^26^. xCell is a gene signatures-based deconvolution method that performs cell-type enrichment analysis across a profile of 64 immune and stromal gene signatures; the output is a cell-type scoring which represents fractional abundance. We applied xCell on all gene expression data and conducted a Wilcoxon rank sum test to assess statistical significance. A p-value of 0.05 was used as the threshold for significance.

### Multiplex immunohistochemistry (mIHC)

Formalin-fixed and paraffin-embedded (FFPE) HGSOC slides from 15 Black and 17 White patients were analyzed using the Opal 7-Color Multiplex IHC kit (AKOYA Biosciences) described previously^55^. Briefly, antigen retrieval was performed in AR9 retrieval buffer (AKOYA Biosciences) on deparaffinized and re-hydrated slides, followed by six cycles of staining procedures including blocking, binding of primary antibodies, second HRP-linked antibodies and visualized with the corresponding Opal fluorophores. Each staining cycle was finished up with heating in AR6 retrieval buffer (AKOYA Biosciences) to release the bounded primary and second antibodies but did not disturb the resident fluorophores. After six-round staining procedures, the slides were counterstained with DAPI. The single marker staining with individual opal fluorophore was employed as the reference for the “spectral unmixing process”. The antibodies and corresponding fluorophores are listed in Supplementary Table 8.

### Acquirement of multispectral images (MSI) and data analysis of mIHC

The Opal fluorophore signals on the stained slides were captured with the Vectra 3 Automated Quantitative Pathology Imaging System (Perkin Elmer) at 200x magnification. Spectral unmixing into four individual fluorophores based on the unique emitting spectrum of each single fluorophore used InForm Advanced Image Analysis software (Akoya Biosciences). Subsequently, the spectral unmixed images underwent cell segmentation based on DAPI, and cell phenotyping based on specific cellular markers through the trained algorithm of Inform. The exported data containing composite images, cell segmentation, and cell phenotyping from InForm were used to quantify cell densities and protein intensities using R-based phenoptrReports & phenoptr (AKOYA biosciences).

### Statistical analysis

Data were analyzed using Student t-test, ANOVA, or Wilcoxon rank sum test. Data are presented as means ± standard deviation (SD), and *P* < 0.05 was considered significant.

## Supplementary information

Supplementary information

## Data Availability

All high-throughput sequencing data and processed data have been deposited in the National Center for Biotechnology Information (NCBI) Gene Expression Omnibus (GEO) data repository: GSEGSE242064. The analyses were performed using publicly available software described in methods.
