# Supplementary material for: Comparative transcriptomic, epigenomic and immunological analyses identify drivers of disparity in high-grade serous ovarian cancer
Source: NPJ Genom Med. 2024 Dec 2;9:64. doi: 10.1038/s41525-024-00448-2 (PMC11612190; doi:10.1038/s41525-024-00448-2)

Supplementary Figures and Tables

Comparative Transcriptomic, Epigenomic and Immunological Analyses Identify Drivers of Disparity in High-Grade Serous Ovarian Cancer

Hao Huang, Russel Keathley, Ujin Kim, Horacio Cardenas, Ping Xie, Jianjun Wei, Ernst Lengyel, Kenneth P Nephew, Guangyuan Zhao, Zhen Fu, Emma L Barber, Masha Kocherginsky, Victoria Bae-Jump, Bin Zhang, Daniela Matei

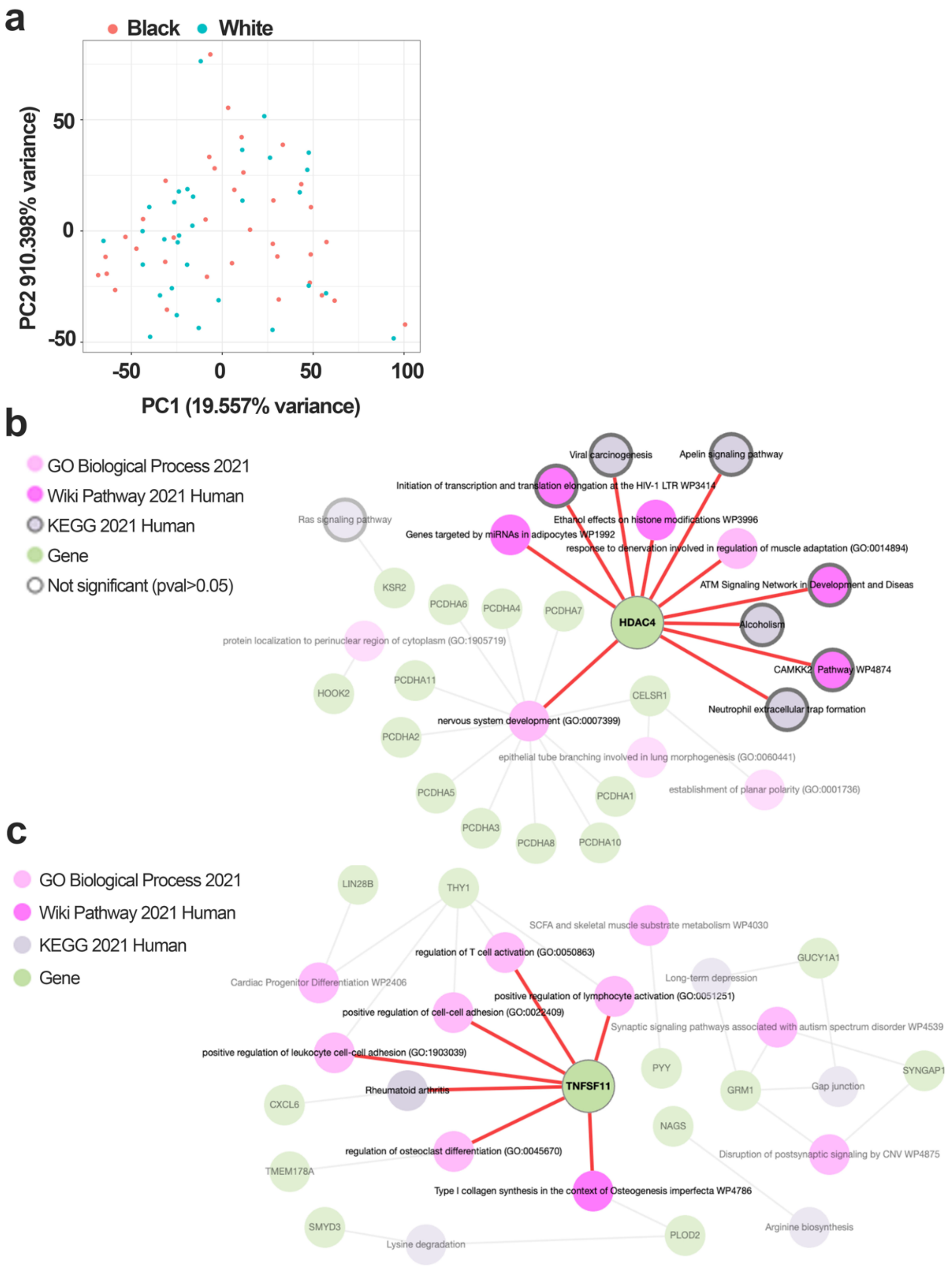

Supplementary Figure 1 Network analysis conducted using three foundational pathway sets (GO Biological Process, KEGG, and Wiki Pathways) reveals connectivity between genes with differential DNA methylation changes and enriched pathways. **a,** PCA of DNA Epic methylation array data from all samples (n = 66 patients). **b,** Pathway enrichment for hypomethylated genes in Black patients using the open website tool Enrichr Knowledge Graph (Enrichr-KG) (https://maayanlab.cloud/enrichr-kg). **c,** Pathway enrichment for hypermethylated genes in Black patients using Enrichr-KG.

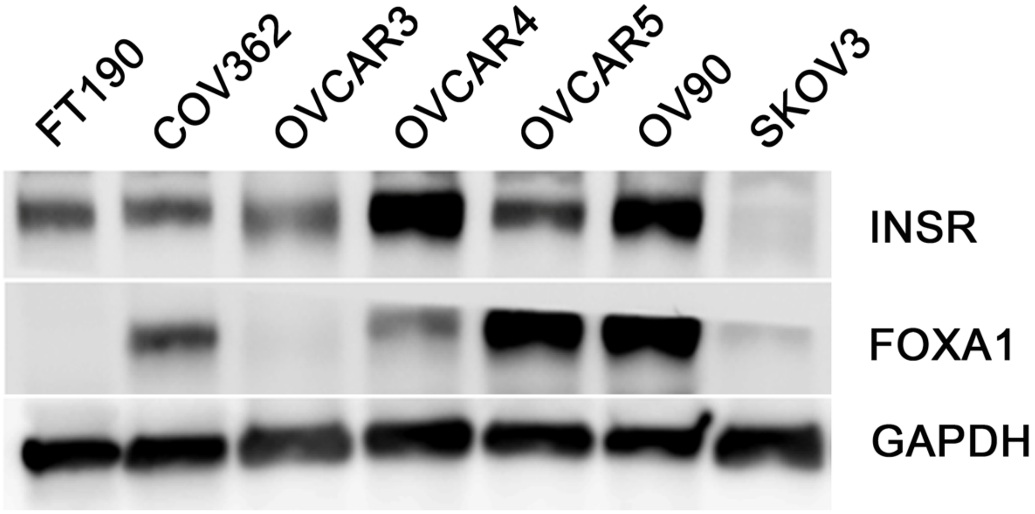

Supplementary Figure 2 Western blot for INSR, FOXA1, and the housekeeping protein GAPDH across OC cell lines and fallopian tube epithelial cells FT190.

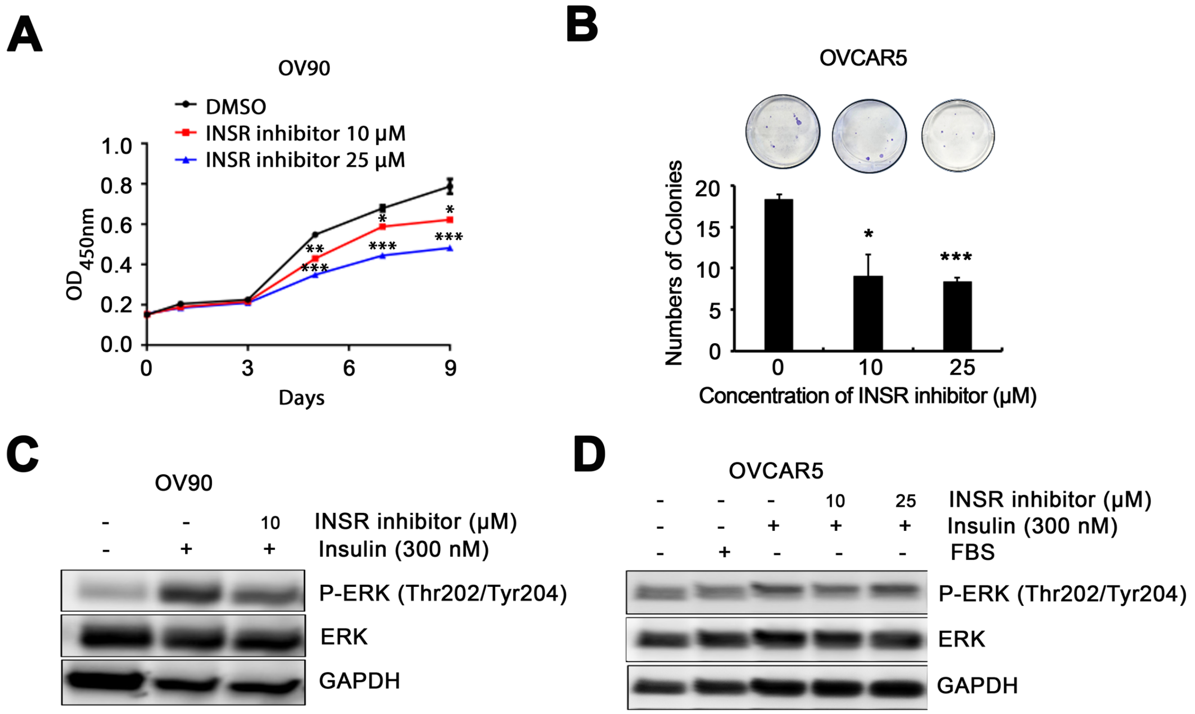

Supplementary Figure 3 Effects of INSR inhibitor (HNMPA-(AM)3) in ovarian cancer cells. **a,** Proliferation assay shows the effects of the INSR inhibitor at 10 and 25 μM on OVCAR5 cells over approximately 10 days. The error bars are defined as mean ± SD, with n =3, * denote $p < 0.05$, ** denote $p < 0.01$, *** denote $p < 0.001$. **b,** Colony-forming assay (CFA) revealed the growth of OVCAR5 cells treated with 10 and 25 μM INSR inhibitor. Bar graph illustrates mean numbers of colonies +/- SEM. * denote $p < 0.05$, *** denote $p < 0.001$ **c, d,** Western blotting illustrates total and phosphorylated ERK after treatment with FBS, insulin, or insulin plus the INSR inhibitor in OV90 cells (c) and OVCAR5 cells (d).

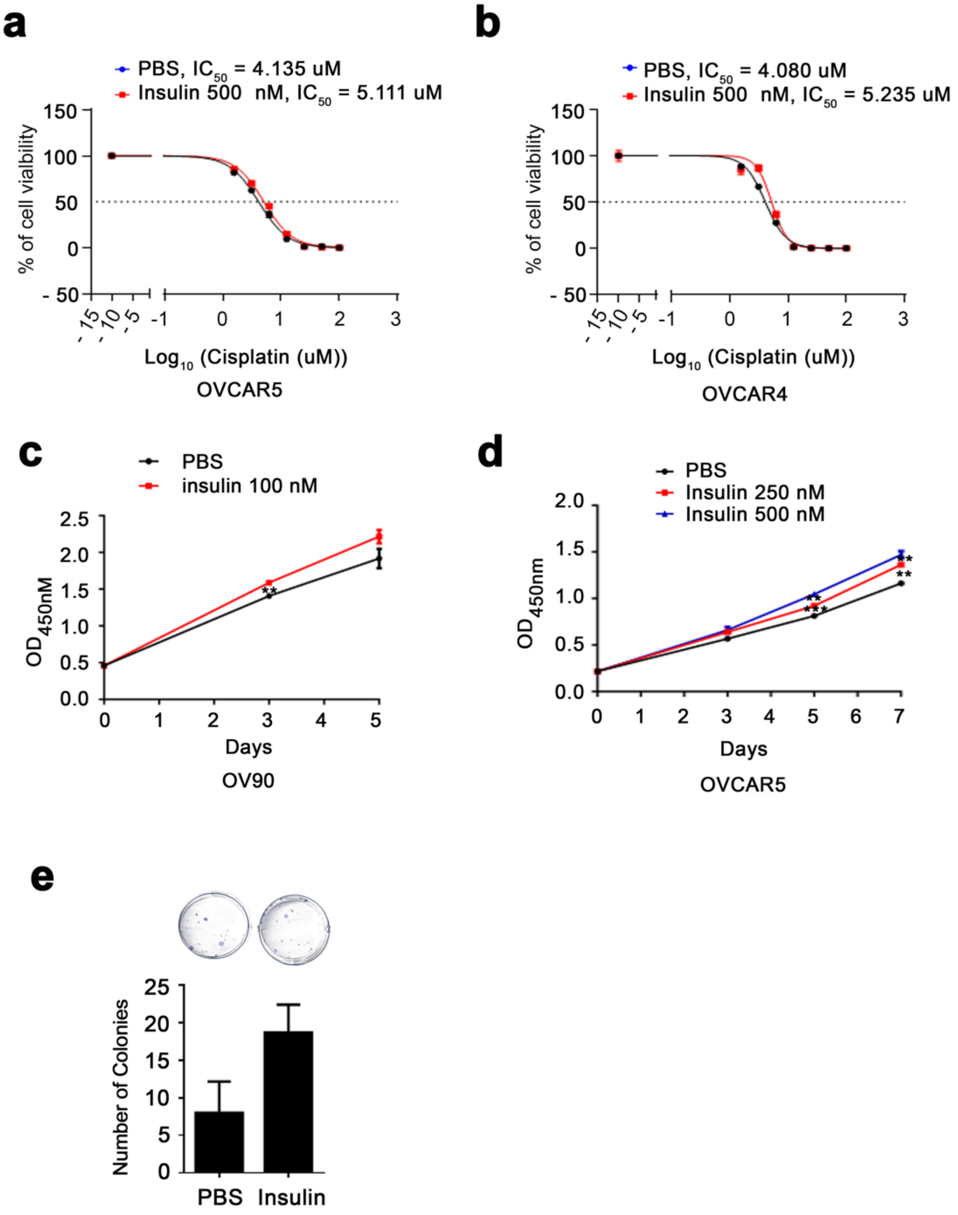

Supplementary Figure 4 Effects of insulin in ovarian cancer cells. **a,** A cell viability assay measured the IC50 for cisplatin response in OVCAR5 cells treated with either 500 nM insulin or PBS, under varying concentrations of cisplatin. **b,** A cell viability assay identified the response of OVCAR4 cells to cisplatin after treatment with either PBS or 500 nM insulin. The IC_{50} values are indicated. **c,** Proliferation assay using a CCK8 assay kit was conducted on OV90 cells treated with 100 nM insulin from 0 to 6 days. The error bars are defined as mean ± SD, with n =3, ** denote $p < 0.01$. **d,** A proliferation assay in OVCAR5 cells treated with two different concentrations of insulin over an 8-day period was performed. The error bars are defined as mean ± SD, with n =3, ** denote $p < 0.01$, ***$p < 0.001$. **e,** A colony-forming assay (CFA) determined the growth of OVCAR5 cells treated with insulin. Quantification is provided below. Bar graph illustrates mean numbers of colonies +/- SEM.

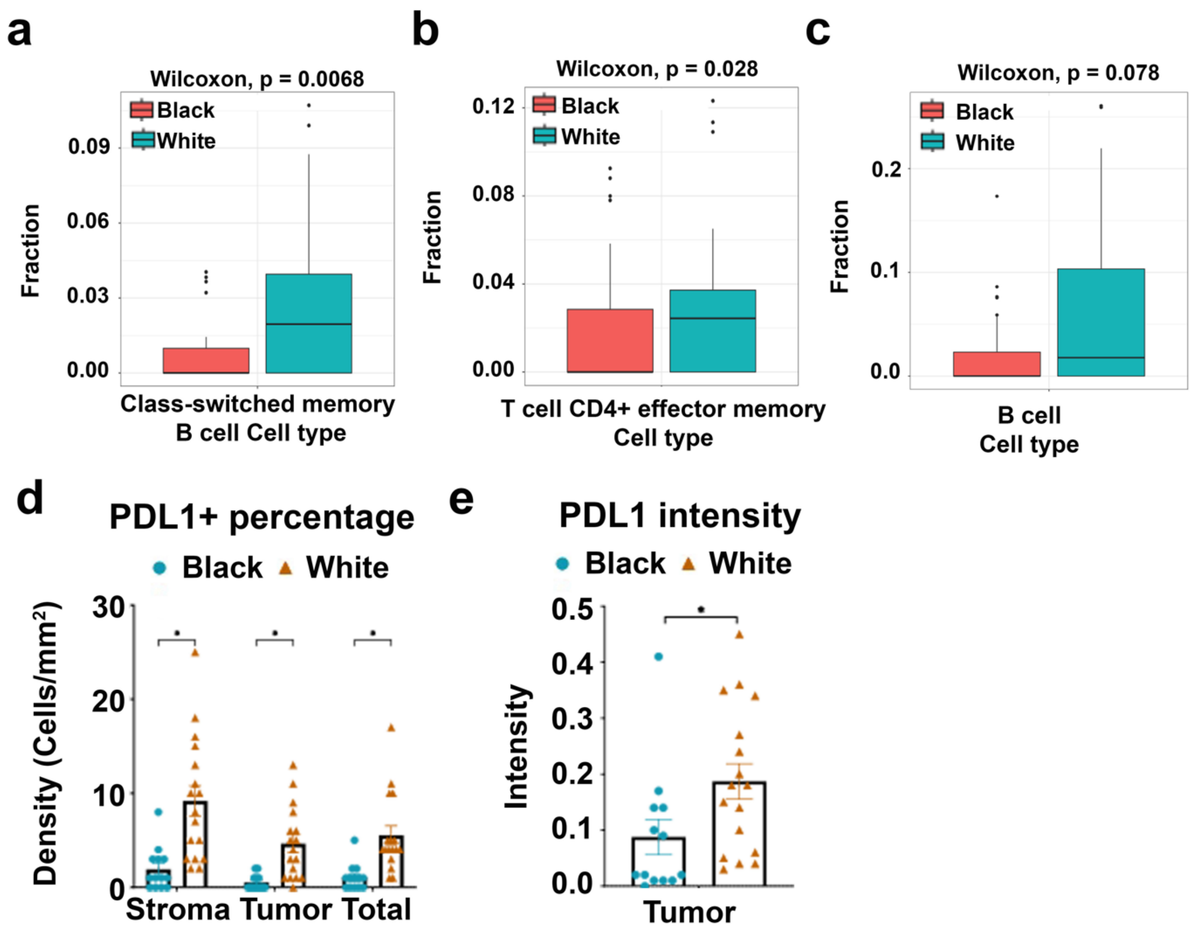

Supplementary Figure 5 Comparative analysis of immune cell populations in Black and White cohorts. **a, b, c,** A bar graph showed the fraction of class-switched memory B cells in Black and White patients (a), Fraction of T cell CD4+ effector memory in Black and White cohorts (b), and fraction of B cells in Black and White cohorts (c). Statistical significance was determined by using the Wilcoxon test, with p-values indicated. **d, e,** PDL1 level was measured by multiplex IHC in stromal and tumor tissues between Black and White ovarian cancer patients. Bar graphs (means ± SE, n=15 and 17, respectively) show the percentage of positive PDL1 in stromal, tumor, and total cell populations (d), and intensity of PDL1 protein in tumor area (e). * denote $p < 0.05$.

Supplementary Table 1. Patients' characteristics

Patients	Source	Race	Histologic subtype	Age	BMI	Mutation	Year of diagnosis,	Stage of disease
W1	NU	White	HGSOC	62	23.61	Negative	2016	IIIC
W2	NU	White	HGSOC	58	28.01	N/A	2017	N/A
W3	NU	White	HGSOC	55	25.09	Negative	2017	N/A
W4	NU	White	HGSOC	68	27.42	Negative	2017	IIIC
W5	NU	White	HGSOC	60	33.25	Negative	2018	IVA
W6	NU	White	HGSOC	76	34.18	Negative	2018	IVB
W7	NU	White	HGSOC	70	25.45	Negative	2018	IIIA
W8	NU	White	HGSOC	63	18.00	Negative	2019	N/A
W9	NU	White	HGSOC	64	29.26	Negative	2019	N/A
W10	NU	White	HGSOC	73	20.65	Negative	2020	IIIC
W11	NU	White	HGSOC	76	27.25	Negative	2020	IIIB
W12	NU	White	HGSOC	61	26.56	Negative	2019	IIIC
W13	NU	White	HGSOC	71	22.89	Negative	2016	IVB
W14	NU	White	HGSOC	72	21.96	Negative	2020	IIIC
W15	NU	White	HGSOC	51	24.23	Negative	2020	IIIC
W16	NU	White	HGSOC	80	26.63	Negative	2020	IIIC
W17	NU	White	HGSOC	52	25.19	Negative	2020	IIIB
W18	NU	White	HGSOC	54	25.54	BRCA2+	2020	IIIB
W19	NU	White	HGSOC	58	21.95	Negative	2021	IIIC
W20	NU	White	HGSOC	70	50.52	Negative	2021	IC3
W21	NU	White	HGSOC	72	27.56	Negative	2021	IIIC
W22	NU	White	HGSOC	60	20.37	BRCA1+	2021	IIB
W23	NU	White	HGSOC	74	37.82	BRCA2+	2021	IIIC
W24	NU	White	HGSOC	54	22.09	Negative	2021	N/A
W25	NU	White	HGSOC	60	34.11	Negative	2021	N/A
W26	NU	White	HGSOC	60	23.48	Negative	2021	N/A
W27	NU	White	HGSOC	82	23.98	Negative	2021	IIIB
W28	NU	White	HGSOC	65	33.84	Negative	2022	IIIC
W29	NU	White	HGSOC	66	18.71	Negative	2021	IIIC
W30	NU	White	HGSOC	50	26.78	BRCA2+	2021	IIIC
W31	NU	White	HGSOC	63	33.42	Negative	2021	IA
B1	UC	Black	HGSOC	65	22.86	BRCA2+	2019	IIIC
B2	UC	Black	HGSOC	62	36.73	RAD 51C	2019	IVB
B3	UC	Black	HGSOC	66	27.81	Negative	2019	IIA
B4	UC	Black	HGSOC	58	29.65	N/A	2019	IIIC
B5	UC	Black	HGSOC	60	42.39	N/A	2019	IIIC
B6	UC	Black	HGSOC	68	28.90	N/A	2013	IIIB
B7	UC	Black	HGSOC	55	36.91	BRCA1	2013	IIIA1
B8	UC	Black	HGSOC	69	19.57	N/A	2013	IVA
B9	UC	Black	HGSOC	89	40.54	N/A	2012	IIC
B10	UC	Black	HGSOC	75	25.97	N/A	2012	IIIC
B11	IU	Black	HGSOC	52	N/A	N/A	N/A	N/A
B12	NU	Black	HGSOC	64	33.17	Negative	2018	IVB
B13	IU	Black	HGSOC	43	N/A	Negative	2017	IB

B14	IU	Black	HGSOC	62	N/A	BRCA1+	2010	N/A
B15	IU	Black	HGSOC	68	N/A	Negative	2010	IIIC (FIGO)
B16	IU	Black	HGSOC	54	N/A	N/A	N/A	N/A
B17	IU	Black	HGSOC	73	N/A	Negative	2016	N/A
B18	IU	Black	HGSOC	53	N/A	N/A	2003	IV
B19	CHC	Black	HGSOC	50	33.23	N/A	2008	IIIC
B20	CHC	Black	HGSOC	63	30.51	N/A	2006	IIIC
B21	CHC	Black	HGSOC	78	22.09	N/A	2008	IIIC
B22	CHC	Black	HGSOC	72	29.53	N/A	2007	IIIC
B23	CHC	Black	HGSOC	73	28.84	N/A	2004	III
B24	CHC	Black	HGSOC	52	25.47	N/A	2003	IVB
B25	CHC	Black	HGSOC	62	22.89	N/A	2009	IIIC
B26	CHC	Black	HGSOC	36	35.89	N/A	2011	IV
B27	CHC	Black	HGSOC	64	36.12	N/A	2011	IIIC
B28	IU	Black	HGSOC	N/A	N/A	N/A	N/A	N/A
B29	CHC	Black	HGSOC	65	25.87	N/A	2008	IIIC
B30	CHC	Black	HGSOC	61	62.86	N/A	2008	IIIC
B31	UNC	Black	HGSOC	44	36.00	Negative	2019	IIIC
B32	UNC	Black	HGSOC	43	27.00	N/A	1997	IV
B33	UNC	Black	HGSOC	68	31.00	Negative	2021	IIIC
B34	UNC	Black	HGSOC	55	32.00	N/A	2013	III
B35	UNC	Black	HGSOC	59	22.00	Negative	2017	IVB

NU: Northwestern University; UC: University of Chicago; IU: Indiana University; UNC: University of North Carolina; CHC: Children's Hospital, Columbus (NRG Oncology). BMI: The body mass index. HGSOC: High-grade serous ovarian carcinoma

Supplementary Table 2. Age and BMI for patients whose tumors were analyzed by RNA-seq and DNA methylation

	**Age**		**BMI**	
	**White**	**Black**	**White**	**Black**
**Mean**	64.52	61.00	27.22	32.67
**Median**	63.00	61.00	26.56	31.00
**Range**	50 ~ 82	36 ~ 89	18.00 ~ 50.52	19.57 ~ 62.86
**# of patients**	31	35	31	27
**T-test**	0.195		0.060	

Supplementary Table 3. List of genes associated with significantly differentially methylated probes in tumors from White vs. Black patients

Probe_ID	Diff. b value	P val_adj	Gene symbol
cg05586384	-0.5793491	0.00603569	RTKN2
rs6426327	-0.3883189	0.01285293	SMYD3
rs6982811	-0.3228529	0.03282394	AC090809.1
rs213028	-0.3226392	0.04086011	ECE1
cg25712921	-0.247587	0.02846044	AC069547.2;SGMS1
cg10654284	-0.0648101	0.01505879	LIN28B
cg21964928	-0.176245	0.04822341	AC026688.1
cg16566400	-0.1667448	0.0335294	THY1;USP2-AS1
cg10708761	-0.1646829	0.03413746	TMEM178A
cg02304226	-0.1348316	0.0070414	AC007608.3
cg16856049	-0.1401251	0.01309313	AC026765.3
cg00555816	-0.091491	0.00570972	MCF2L
cg12087371	-0.110186	0.0256779	CXCL6
cg22234419	-0.132927	0.02041661	PLOD2
cg01777861	-0.1027179	0.00389672	AC021220.2
cg01290791	-0.1310233	0.01008587	LINC01250
cg00693994	-0.014974	0.00100496	GUCY1A1
cg03478199	-0.0974719	0.01136249	GRM1
cg01128482	-0.1106736	0.00641514	HOXD3
cg10304824	-0.0982653	0.00641514	HAGLR;HOXD3
cg03733219	-0.0713332	0.00091347	SPRED3
cg00919398	-0.1100047	0.00115287	SVOPL
cg01357135	-0.0962381	0.00647833	PPM1L
cg00332153	-0.0961865	0.01502427	BANK1
cg05180443	-0.0797915	2.4505E-05	ACSF2;CHAD
cg02652579	-0.1078074	0.02756402	CUTA;SYNGAP1
cg02518216	-0.0850371	0.02773363	NUAK1
cg03186333	-0.0937889	0.01053041	EYA1
cg04032226	-0.0892064	1.8155E-05	NAGS;PYY
cg09745430	-0.0730596	0.04830955	AL132996.1;BACH2
cg01250603	-0.0799008	0.03859027	AC010998.1
cg04536296	-0.0919352	0.00471917	TNFSF11
cg06088084	-0.0132829	0.03434901	BMS1P1;GLUD1P2
cg01422243	-0.0765988	0.00305797	SPAG6
cg07655045	-0.0564927	0.00385099	AC010624.1;AC010624.3;ZNF473
cg07542810	-0.0798294	0.00989037	AC010457.1
cg11186858	0.1161596	0.04566027	SEC14L1

cg02898051	0.11627631	0.04495608	AC025283.2
cg07673080	0.11789605	0.042711	HDAC4
cg07599507	0.12905023	0.02426188	CSMD3
cg26161709	0.1021996	0.02426188	RNU4-37P
cg07363697	0.15639785	0.00950913	LINC02684
cg02207052	0.14857439	0.00053575	CELSR1
cg04598224	0.1269318	0.01736086	AC008543.1
cg01227835	0.16479338	0.03537051	AC000372.1
cg08067695	0.14178413	0.00492101	PCDHGA1;PCDHGA2;PCDHGA3;PCDHGA4;PCDHGA5;PCDHGB1; PCDHGB2
cg00598449	0.15438901	0.02246887	ARHGEF38
cg09852127	0.17885926	1.8139E-05	PCDHA1;PCDHA10;PCDHA11;PCDHA12;PCDHA2;PCDHA3;PCDHA4; PCDHA5;PCDHA6;PCDHA7;PCDHA8;PCDHA9
cg13295463	0.14003023	1.8139E-05	AC005609.3;PCDHA1;PCDHA10;PCDHA11;PCDHA12;PCDHA2; PCDHA3; PCDHA4;PCDHA5;PCDHA6;PCDHA7;PCDHA8;PCDHA9
cg12945378	0.16136908	0.01467474	KSR2
cg04657146	0.17991003	0.02452639	HOOK2
rs10882854	0.37429222	0.00710803	LCOR

Supplementary Table 4. List of top 20 upregulated genes in tumors from Black vs White patients.

Gene symbol	Fold change	*P* value	FDR
TBC1D3D	3.873	1.36E-06	0.00067
MAGEC2	3.762	9.82E-05	0.015673
FOXB1	3.596	6.74E-07	0.000422
MLC1	3.277	3.16E-09	4.45E-06
MUC5AC	3.272	1.20E-07	9.31E-05
RHBG	3.215	5.10E-08	4.41E-05
MUC5B	3.129	6.09E-07	0.000392
JPH3	2.858	1.06E-08	1.19E-05
EEF1A2	2.807	1.04E-08	1.19E-05
SHISA3	2.801	1.21E-06	0.00065
CSF3	2.701	0.000128803	0.018451
ADGRA1	2.619	7.27E-05	0.013199
TOX3	2.559	0.000310732	0.032686
EDN3	2.491	0.000356273	0.035645
PADI1	2.472	0.000184109	0.023284
CHAT	2.438	3.60E-05	0.009319
FOXA1	2.427	0.000168391	0.022039
MB	2.426	1.05E-05	0.003387
NPIPA8	2.406	1.28E-06	0.000668
NOTCH2NLB	2.401	1.94E-09	3.12E-06

Supplementary Table 5. List of top 20 down-regulated genes in tumors from Black vs White patients.

Gene symbol	Fold change	*P* value	FDR
FDCSP	-4.936	2.39E-09	3.59E-06
GAGE13	-4.822	0.000127	0.018451
SEZ6	-4.330	1.38E-17	5.16E-14
CTAG1B	-3.860	9.14E-06	0.003185
CNMD	-3.672	4.45E-05	0.010139
SOX3	-3.646	0.000117	0.017807
NPB	-3.548	1.87E-11	3.51E-08
KEL	-3.412	2.98E-13	6.71E-10
HOXD11	-3.281	6.70E-06	0.002557
NXF3	-3.009	3.45E-09	4.56E-06
HOXD13	-3.008	0.000297	0.03245
MAGEC1	-2.972	0.00034	0.034796
CRYGD	-2.942	0.000417	0.039806
BCAN	-2.721	4.76E-06	0.00188
CD1A	-2.696	3.59E-06	0.00147
NPHS1	-2.694	1.20E-06	0.00065
CDH12	-2.675	1.67E-05	0.004636
OR2I1P	-2.526	2.58E-06	0.001117
CPNE4	-2.465	1.37E-06	0.00067
PAX5	-2.454	1.49E-05	0.004302

Supplementary Table 6. Patients' characteristics for tumors included in the TMA used for validation

Source	Race	Age	Histologic subtype	Stage of disease
NU	Black	46	HGSOC	IV
NU	Black	63	HGSOC	IVA
NU	Black	64	HGSOC	IVB
NU	Black	83	HGSOC	IVA
NU	Black	50	HGSOC	IVA
NU	Black	62	HGSOC	IIIC
NU	Black	73	HGSOC	IIIB
NU	Black	69	HGSOC	IIIC
NU	Black	63	HGSOC	IIIC
NU	Black	68	HGSOC	IIIC
NU	White	71	HGSOC	IVB
NU	White	62	HGSOC	IIIC
NU	White	58	HGSOC	IIIA
NU	White	53	HGSOC	IIIC
NU	White	60	HGSOC	IVA
NU	White	65	HGSOC	IVB
NU	White	76	HGSOC	IVB
NU	White	64	HGSOC	IIIB
NU	White	51	HGSOC	IIIC
NU	White	80	HGSOC	IIIC
NU	White	52	HGSOC	IIIB
NU	White	54	HGSOC	IIB
NU	White	58	HGSOC	IIIC
NU	White	70	HGSOC	IC3
NU	White	71	HGSOC	IIIC
NU	White	60	HGSOC	IIB
NU	White	74	HGSOC	IIIC
NU	White	60	HGSOC	IVB
NU	White	52	HGSOC	IIIC
NU	White	82	HGSOC	IIIB
NU	White	63	HGSOC	IA
NU	White	74	HGSOC	IIIA2

NU: Northwestern University; HGSOC: High-grade serous ovarian carcinoma.

Supplementary Table 7. List of primers sequences

Target gene	Forward primer (5'to 3')	Reverse primer (5'to 3')
18S	ACCCGTTGAACCCCATTCGTGA	GCCTCACTAAACCATCCAATCGG
FOXA1	CTACTCGTACATCTCGCTCATC	GCTGGTTCTGCCGGTAATA
FOXB1	TACATCTCGCTGACCGCTAT	TGCGTGTTCTCCCTGTAGTA
EEF1A2	ATCGTGGGCGTGAACAAA	GGTTGTAGCCGATCTTCTTGAT
LDLR	CTCCCGCCAAGATCAAGAAA	GTTTGGAGTCAACCCAGTAGAG
SCD1	ACGATATCTCTAGCTCCTATACC	GGCATCGTCTCCAACTTATC
INSR	CCGGAAGTTACGTCTGATTC	GTGATGGTGAGGTTGTGTT
NXF3	GGATACCTCCAGCAACATAAA	GGGCTTCAGTACCACATAAA
CPNE4	CCTATCAGAGCTGTCTTCCTA	CCTCCTTGGTGTTAGTTTCC
WNT16	CAGTTCAGACACGAGAGATG	CAGCCATCACAGCATAAATAAA

Supplementary Table 8. Antibodies used for multiplex IHC

Antigen	Dilution	Venders	Catalog #	Fluorophore
CD3	5x	Biocare medical	PP215AA	Opal 650
CD4	1x	Biocare medical	104R-27	Opal 520
CD8	200x	Cell signaling	70306S	Opal 540
FOXP3	100x	BioLegend	320102	Opal 570
PD-L1	100x	Cell signaling	13684S	Opal 620
PanCK	200x	abcam	ab7753	Opal 690

Supplementary Material

Unedited full blot images for Figure 4b

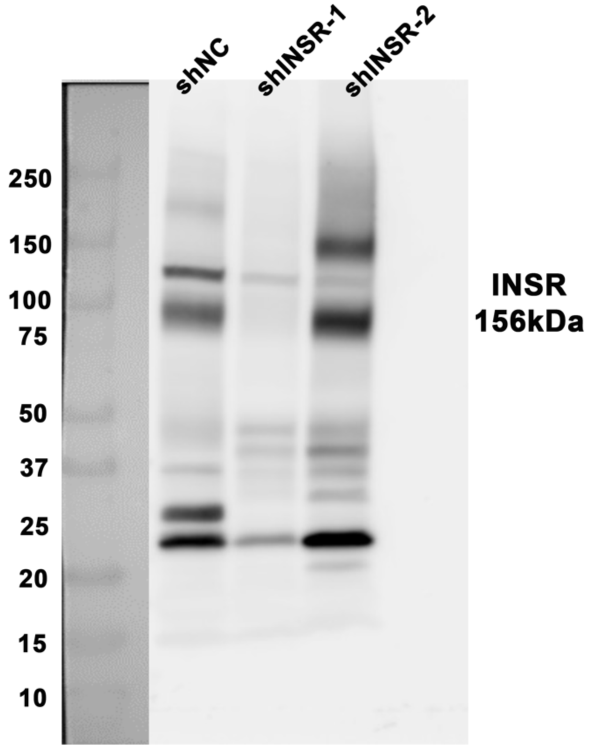

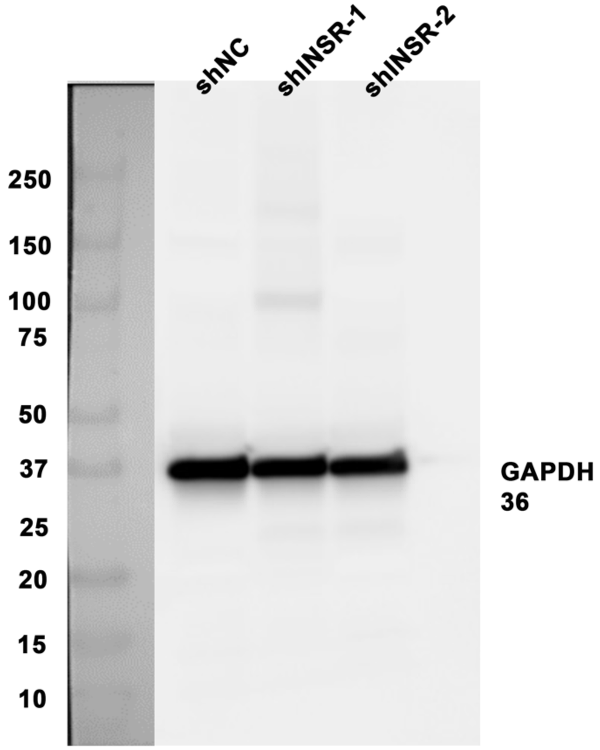

Unedited full blot images for Figure 5b

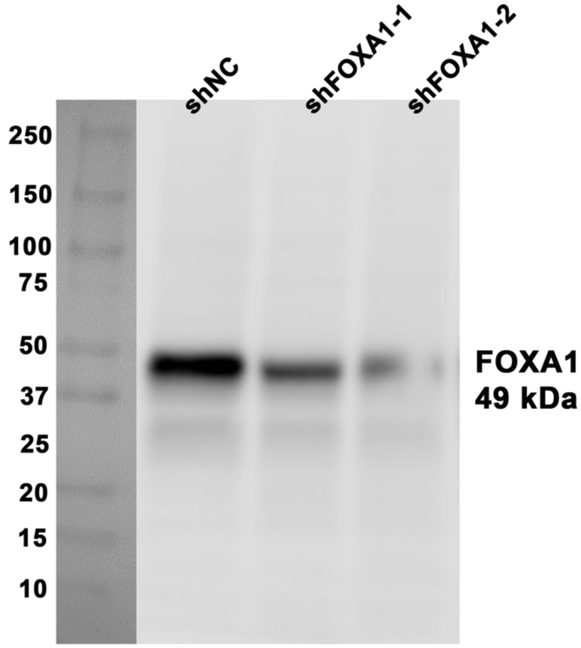

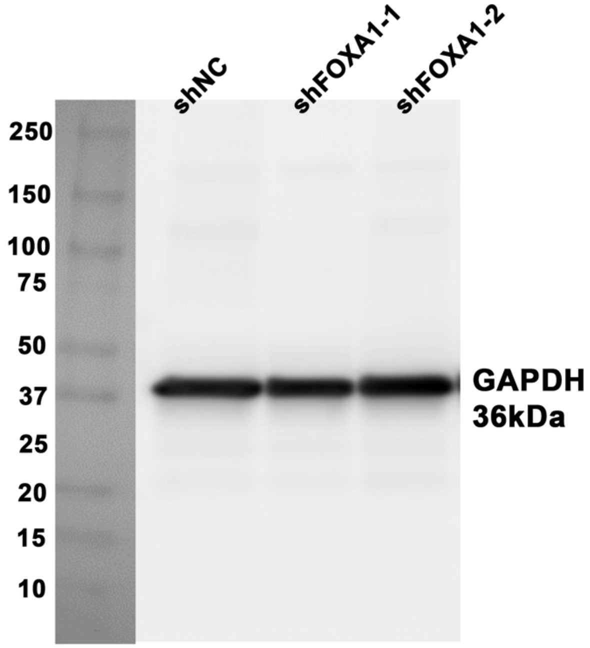

Unedited full blot images for supplementary Figure 2

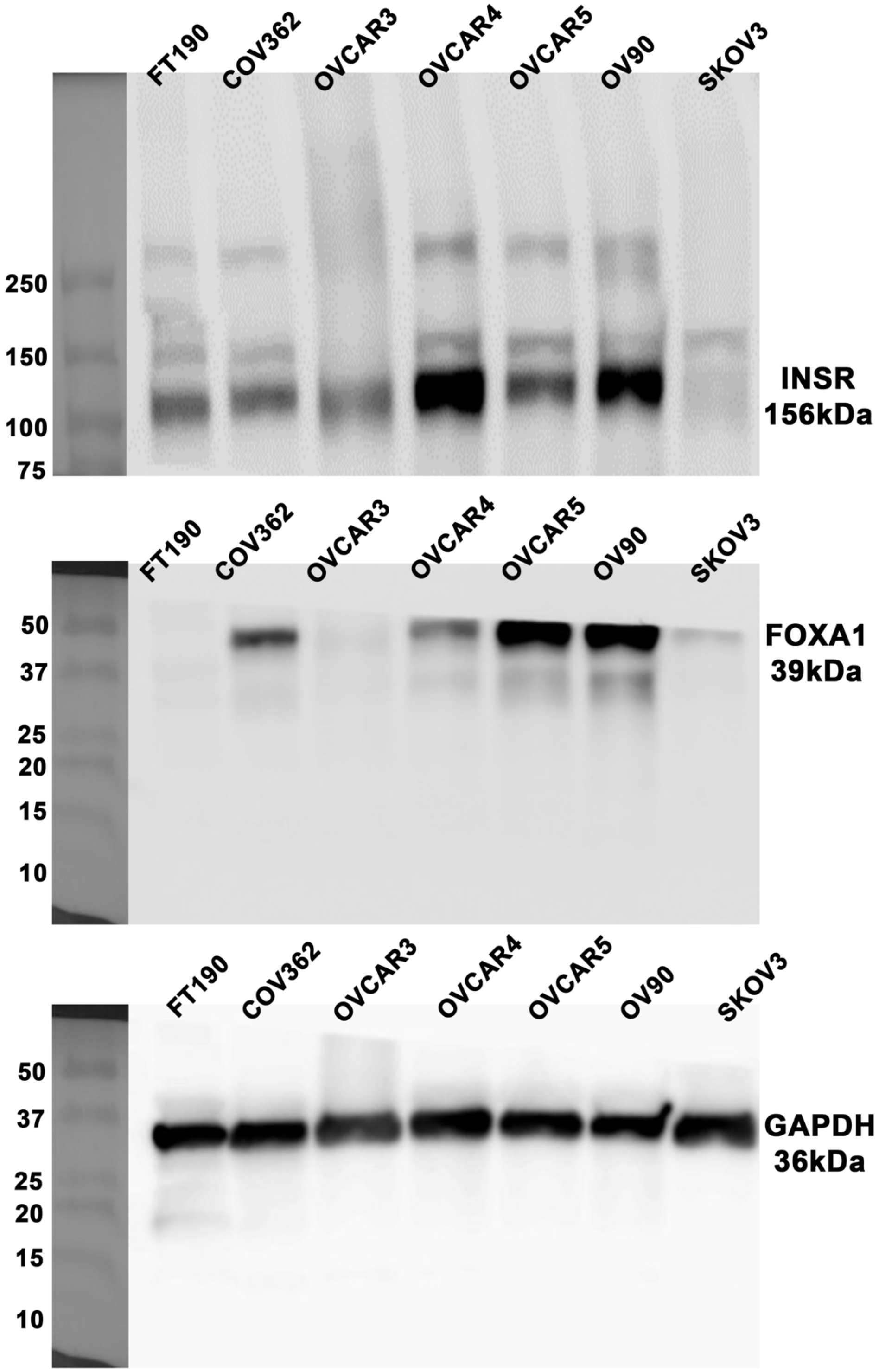

Unedited full blot images for supplementary Figure 3c

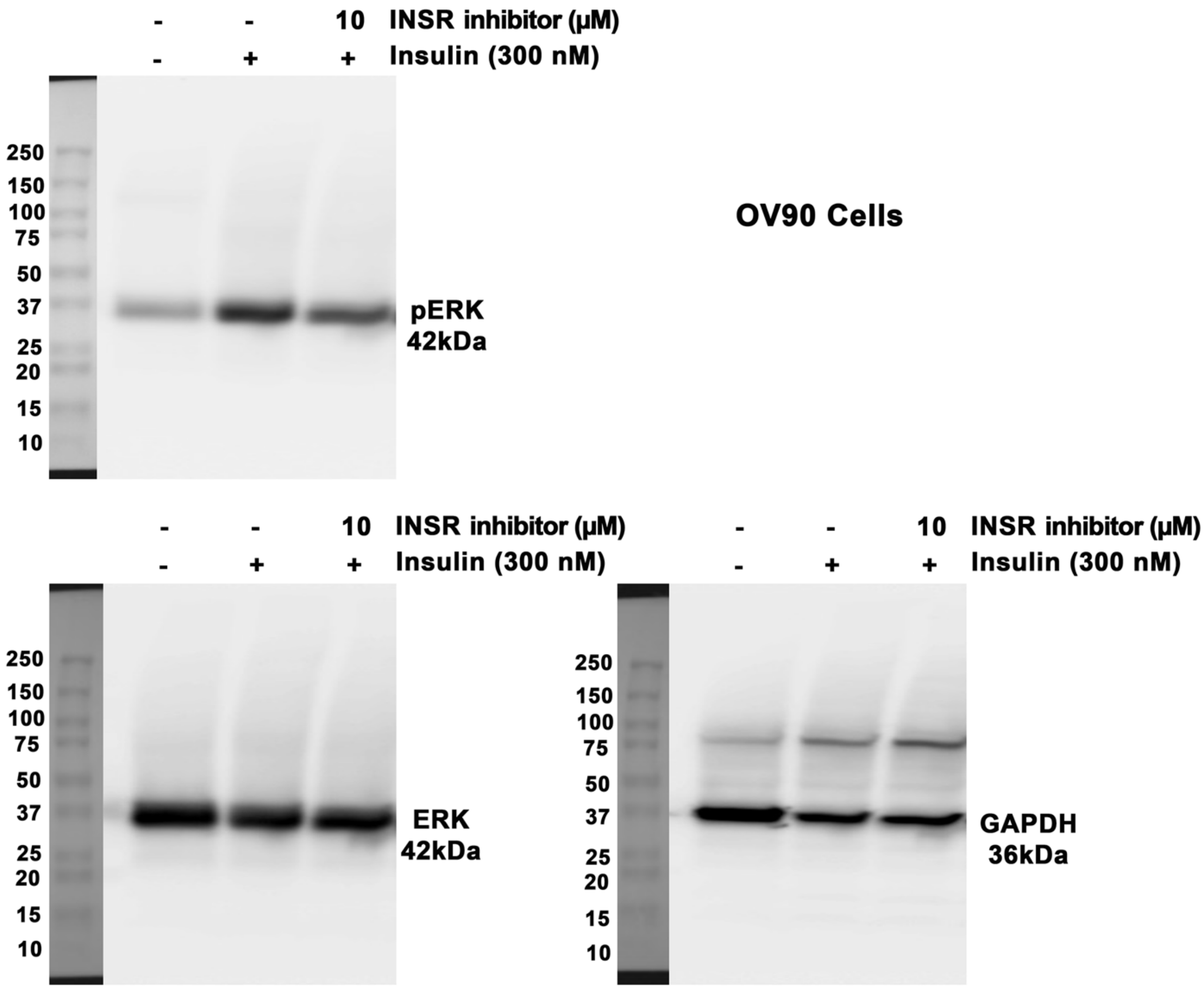

Unedited full blot images for supplementary Figure 3d

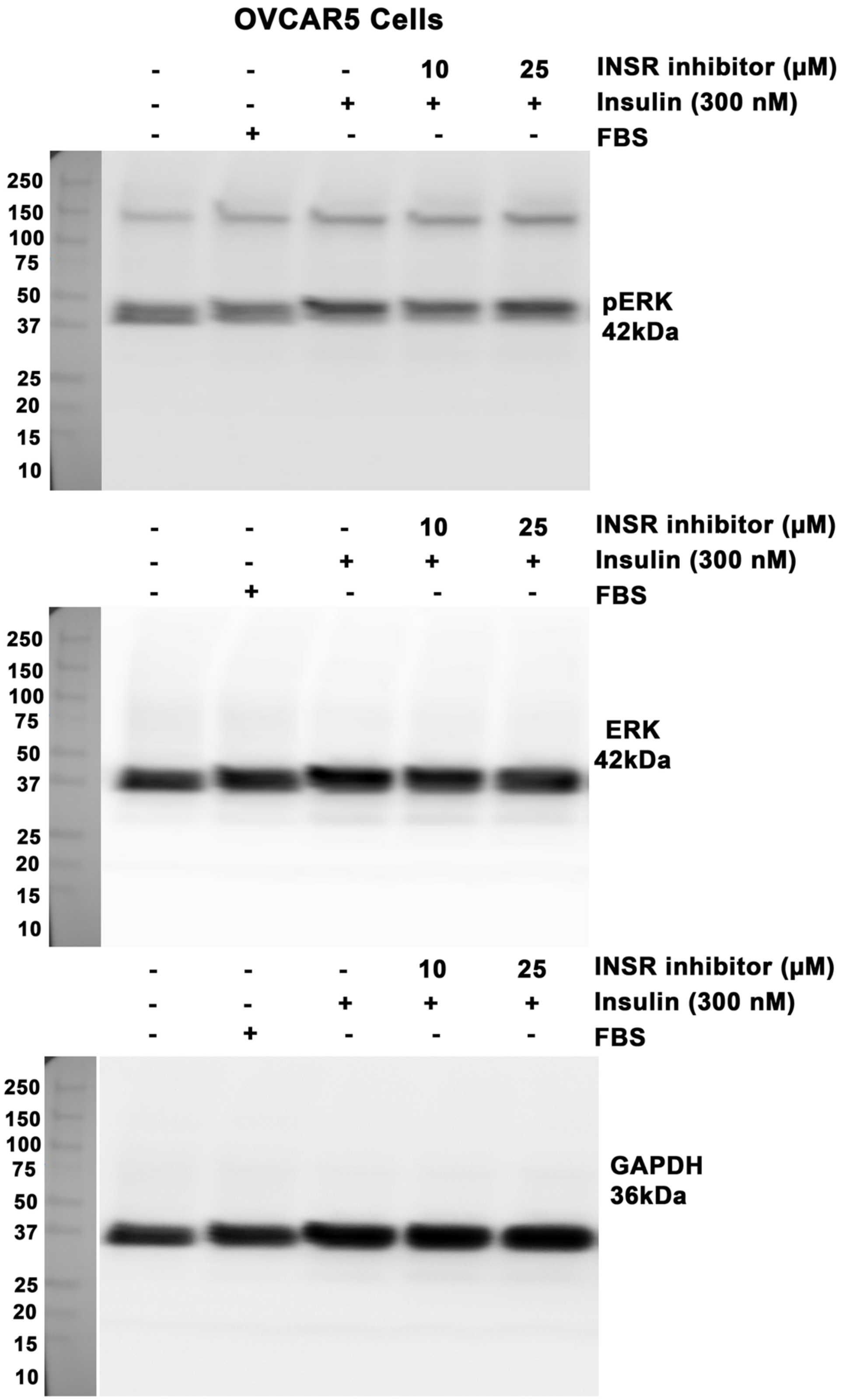

Supplement: Supplementary file 1 — Supplementary information [file 41525_2024_448_MOESM1_ESM.pdf]
